# Large-scale data reveal disparate associations between leisure time physical activity patterns and mental health

**DOI:** 10.1038/s43856-023-00399-2

**Published:** 2023-12-21

**Authors:** Ying Zhou, Chenshuang Li, Wei Wang, Lieyun Ding

**Affiliations:** 1 Center for Smart and Healthy Buildings, Huazhong University of Science and Technology, 430074 Wuhan, Hubei China; 2 Department of Neurology, Tongji Hospital, Huazhong University of Science and Technology, 430030 Wuhan, Hubei China

**Keywords:** Public health, Epidemiology

## Abstract

**Background:**

Leisure time physical activity (LTPA) is known to be associated with a lower risk for mental health burden, while whether the underlying mechanisms vary across populations is unknown. We aimed to explore the disparate associations between LTPA and mental health based on large-scale data.

**Methods:**

In this study, we analyzed data including 711,759 individuals aged 15 years or above from the latest four rounds (2003, 2008, 2013, and 2018) of the National Health Service Survey (NHSS) in China. We used multiple logistic regression models adjusted for potential confounders to investigate associations between LTPA and mental health in the total population and subgroups by measuring a diverse set of activity frequencies, intensities, and types. To examine the dose-response associations between total activity volume and mental health, we conducted restricted cubic splines to investigate possible nonlinearity.

**Results:**

LTPA was associated with remarkably lower self-reported mental health burden (OR 0.56, 95% CI 0.54–0.58). The dose-response relationship between total activity volume and mental health was highly nonlinear (*p* < 0.001), presenting L-shaped with first 1200 metabolic equivalents of task (METs)-min/week for significant risk reduction (OR 0.58, 95% CI 0.56–0.60). Notably, merely exercising 3–5 times per week with moderate swimming was significantly associated with lower mental health burden among younger people, while the association was strongly large in older adults aged 60 years or above doing 55-min moderate apparatus exercise at least six times a week.

**Conclusions:**

In a large Chinese sample, LTPA was meaningfully and disparately associated with mental health burden across different people. Policy targeted at prompting activity may be effective for reducing mental health burden, but importantly, tailored strategies are needed based on population contexts.

## Plain language summary

Physical activity that is undertaken as a leisure activity is known to be beneficial for mental health. It is unclear whether the reasons for this vary across different populations. We studied more than 700,000 people living across China. We found that the frequency, duration, type, and intensity of physical leisure activity and their association with a person’s mental health vary depending on gender, age, lifestyle and presence of chronic disease conditions. The best mental health was found in those who did not exercise a lot or very little. Notably, exercising 3–5 times per week by swimming at a moderate pace was significantly associated with lower mental health burden among younger people, whilst for those aged over 60 it was most beneficial to do 55-min moderate apparatus exercise at least six times a week. Our study highlights that prompting activity may be effective for reducing mental health burden and that guidance should be specific for particular age groups.

## Abstract (web summary)

Zhou et al. analyze leisure time physical activity and self-reported mental health in people of different ages in China. Exercise is associated with better mental health, even if exercise is of moderate intensity.

## Introduction

In the last decade, the mental health burden has increased by nearly 13% worldwide, which contributes to poor health outcomes and large economic losses^1–3^. The outbreak of the COVID-19 pandemic has further exacerbated many determinants of poor mental health, such as depressive and anxiety disorders^4–8^. Leisure time physical activity (LTPA) as a low-cost approach has been proven to have positive effects on mental health benefits to help reduce depression, anxiety, and emotional problems^9–17^.

However, evidence to date on LTPA and mental health provided conflicting results^15–17^. It has been advocated in some studies that a higher activity level is better^15,16^, while some studies suggested negative psychological effects from a high level of activity^17^. These conflicting results might be potentially attributed to the use of non-representative or small samples, limited study populations and differences in assessments^15–17^. Moreover, although the dose-response association was widely recognized in activity and mortality^18–20^, none of the current studies have robustly examined the dose-response association between total activity volume and mental health. Usually, activity assessment using binary categorization (low vs high) leads to a loss of information and the variation in mental health risk across a range of activity doses is unknown^14^. Additionally, there is limited evidence on the relationships between specific activity features such as frequency, duration, intensity, and types and mental health. It is still unclear how these associations vary across a range of frequencies or durations and different intensities or types. Third, because of the specificity and heterogeneity of different people^14,21^, the association between different activity forms and mental health can vary across populations. Identifying what format of activity will best benefit specific people with mental health problems can inform making personalized recommendations and help develop and implement cost-effective interventions. Therefore, considering these critical research gaps, there is a pressing need to understand the disparate associations between a diverse set of activity features and mental health burden across various individuals.

In this article, we contribute to the research literature on mental health and physical exercise by analyzing the large-scale sample of 711,759 Chinese individuals. Mental health burden considered in our study includes several types of conditions such as stress, depression, and problems with emotions. We provided a comprehensive examine of the association between LTPA and mental health burden, including quantifying the dose-response relationship, measuring patterns of activity for a diverse set of frequencies, durations, intensities, and types, and further identifying best activity patterns for various people related to lower mental health burden. This study constitutes the largest study of its kind to date, which would be important for informing stakeholders to develop cost-effective strategies. In our study, we found that associations between frequency, duration, type, and intensity of LTPA and mental health vary in populations across gender, age, lifestyle behaviors, and chronic disease conditions. The dose-response relationship between total activity volume and mental health was highly nonlinear and presented L-shaped with a threshold of 1200 metabolic equivalents of task (MET)-min of activity a week. Notably, exercising 3–5 times per week with moderate swimming was significantly associated with lower mental health burden among younger people, while the association was strongly large in older adults aged 60 years or above doing 55-min moderate apparatus exercise at least six times a week.

## Methods

### Study design and dataset description

Our analyses are based on the latest four rounds (2003, 2008, 2013, and 2018) of the National Health Services Survey (NHSS) (Supplementary Methods and Supplementary Fig. 1). The NHSS was carried out by the National Health Commission (NHC) of the People’s Republic of China covering all 31 provinces, autonomous regions, and municipalities in the mainland of China, using multistage stratified cluster sampling method (Supplementary Methods and Supplementary Fig. 1)^22,23^. The institutional review board of the Chinese National Bureau of Statistics provided approval of the survey. Individuals aged 15 years or above were eligible for the survey and oral consent was obtained from all respondents before the interview. The previous report has presented the interview procedures of NHSS in detail^22,23^. Based on a structured questionnaire, face-to-face interviews were conducted by local trained healthcare workers including information about participants’ demographic, socioeconomic, lifestyle behaviors (e.g., alcohol consumption, smoking, and LTPA), and health conditions (Supplementary Data 1). More details of the study design and data collection process can be seen in Supplementary Information (Supplementary Methods, Supplementary Figs. 1 and  2).

### Outcome variable

Following previous research in the measurement of mental health burden^17^, we defined mental health burden as an assessment protocol for experience of probable problems in mental health in the past 30 days in NHSS. Mental health burden was measured with participants’ self-report question: “Now thinking about your mental health, which includes stress, depression, anxiety, and problems with emotions, whether you have these problems during the past 30 days was your mental health not good? (no problems, moderate problems, and severe problems)” The criteria for participants who had mental health burden included those who had experienced moderate or severe problems, or any combination thereof in mental health. A binary outcome variable comprised individuals without mental health burden (0 or “no case”) and individuals who had experienced mental health burden (1 or “case”).

### Independent variable

To assess LTPA, we focused on 4 questions including the frequency, duration, type and intensity (Supplementary Data 1). We first asked respondents how often they do LTPA every week in the past 6 months. If the frequency was once or more than once a week, we asked them about the duration of the average session each time (minutes). The respondents were also asked to report details for the types (tai chi, yoga, trotting or jogging, apparatus exercise, aerobics, ball games, dancing, and swimming), as well as the intensity (light; moderate; vigorous). The characteristics of activity features of participants were presented in Supplementary Table 1. We assigned the metabolic equivalent of task (MET) for different intensities of activity and calculated the total volume of activity by multiplying the reported frequency by the duration and intensity (MET)^24^. The total volume of activity was expressed in MET-min per week (MET-min/week) and further categorized as inactive (having no moderate or vigorous-intensity activity), insufficiently active (having some activity but less than 600 MET-min/week), and active (600 MET-min/week or above) in accordance with current guidelines from the American College of Sports Medicine^25^ and the US Centers for Disease Control and Prevention^26^ for adults aged 18 years or above. For adolescents aged 15–17, active was defined according to the guidelines as doing moderate- to vigorous-intensity activity more than 60 min daily^25,26^.

### Covariates

Age was calculated according to birth date and classified into three groups (15—29 years, 30–59 years, and 60 years or above). Gender was expressed as a binary variable (men or women). Smoking was defined as one had smoked a total of at least 100 cigarettes and had not quit smoking and smoking status was dichotomized as “smoker” or “non-smoker”. Alcohol consumption was defined as having had an alcoholic drink in the 12 months before the survey and was dichotomized as “drinker” or “non-drinker”. Income level was classified into three groups (low, middle, and high) based on annual per capita income in the sampled county in the survey year. Education level was categorized into four groups (college or above, high school, middle school, and primary school or below). Occupation status was classified into four groups (employed, retired, student, and unemployed). Marital status was categorized into four groups (married, unmarried, divorced, and widowed). Urbanization (urban or rural) and geographical regions (east, central, or west) were considered on the basis of residential address. Year (2003, 2008, 2013, and 2018) was also adjusted in all models.

### Statistical analysis

To explore the association between LTPA and mental health burden, we conducted multiple logistic regression models. We explored the association between activity frequency, type, and intensity and mental health burden using the regression models not only for all people but also for specific groups. The regression models were adjusted for covariates, including gender, age, education, income, occupation, marital status, smoking, alcohol consumption, geographical regions, urbanization, and year. The results were presented with odds ratios (ORs) and 95% confidence intervals (CIs). We applied restricted cubic splines to investigate possible nonlinearity between activity duration and total volume activity with mental health and the model was also adjusted for aforementioned covariates. The analyses used all pooled data from the four surveys. Individuals with missing values were excluded in the final analysis. All analyses used Stata 16.0 or R 3.5.1.

### Reporting summary

Further information on research design is available in the Nature Portfolio Reporting Summary linked to this article.

## Results

The NHSS sampled 57,023 households from 95 counties in 2003, 56,456 households from 94 counties in 2008, 93,613 households from 156 counties in 2013, and 94,076 households from 156 counties in 2018. A total of 711,759 individuals, including 150,568 in 2003, 139,987 in 2008, 216,005 in 2013, and 205,119 in 2018, were analyzed in the study (Supplementary Fig. 2). Notably, 7.92% of individuals had self-reported mental health burden in China (Supplementary Table 2). Compared with inactive people (8.75%), active individuals had a lower percentage of mental health burden (5.56%) (Supplementary Fig. 3 and Supplementary Data 2).

### Associations between different forms of activity with mental health in total population

**Fig. 1:**
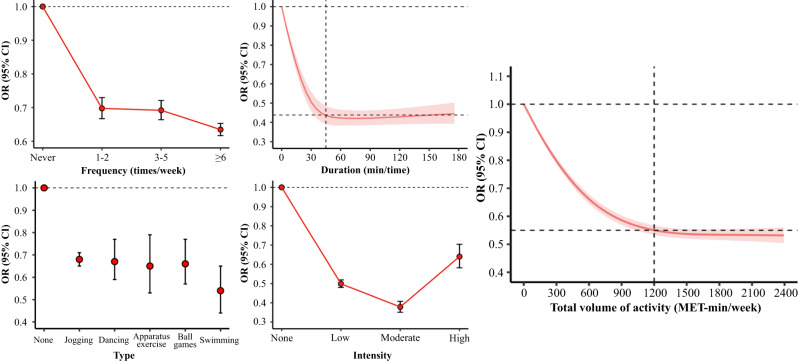
The associations between different forms of leisure time physical activity with mental health in the total population (*N* = 711,759). Models were adjusted for geographical regions, urbanization, gender, age, marital status, education, employment, income, smoking, alcohol consumption, and year. Bars and ribbons show 95% CIs. OR odds ratio, MET metabolic equivalent of task.

Figure 1 and Supplementary Data 3 present the association between different activity forms with mental health in the total population. The increasing frequency was associated with decreasing mental health burden and those who did activity six times or above a week presented the lowest burden (OR 0.63, 95% CI 0.62–0.65). The L-shaped smoothed regression spline showed that the duration between 45 min and 55 min (peaking around 50 min) was related to the lowest mental health burden (Fig. 1). Although all types of activity were associated with reduced mental health burden, swimming (0.54, 0.44–0.65) and apparatus exercise (0.65, 0.53–0.79) were associated with lower mental health burden compared with others (Fig. 1). Those who engaged moderate activity (0.38, 0.35–0.41) had the maximum reduction in mental health burden compared with light (0.50, 0.48–0.52) or vigorous (0.64, 0.58–0.70) activity. Additionally, the restricted cubic spline presented a significant nonlinear and L-shaped relationship between mental health burden and total volume of activity. As shown in Fig. 1, the mental health burden drops sharply with the activity volume increases and the burden declines slightly after 1200 MET-min/week is achieved.

### Associations between LTPA with mental health in different people

**Fig. 2:**
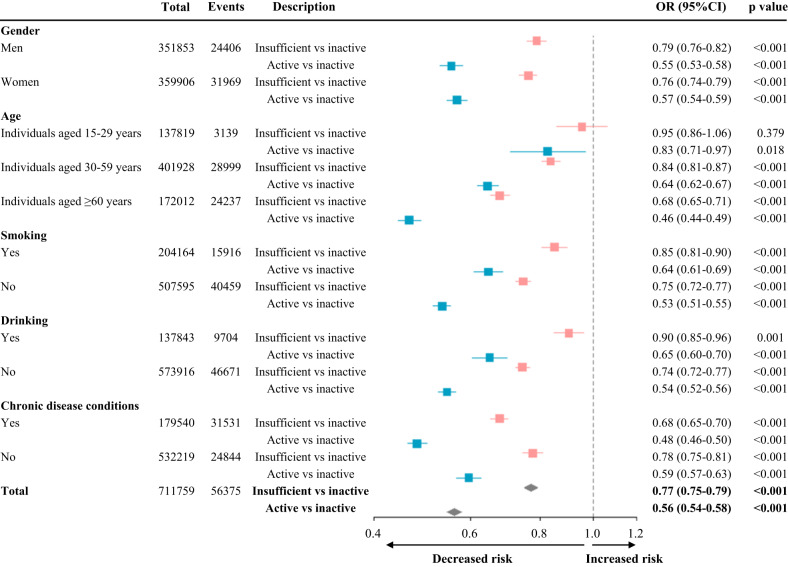
The associations between leisure time physical activity with mental health in different people (*N* = 711,759). Models were adjusted for geographical regions, urbanization, gender, age, marital status, education, employment, income, smoking, alcohol consumption, and year. Chronic diseases must be diagnosed by doctors and defined as having a newly diagnosed chronic condition in the past six months or having a diagnosed chronic condition before six months but suffering from disease symptoms and receiving treatments during the past six months. Inactive (no) is the reference group. Insufficient active = 0–600 MET-min per week. Active ≥ 600 MET-min per week. Bars show 95% CIs. Red bars indicate values of compared groups between active and inactive individuals, and blue bars indicate values of compared groups between insufficient active and inactive individuals. OR odds ratio, MET metabolic equivalent of task. Exact *p*-values for this figure can be found in Supplementary Data 4.

When compared with inactive individuals, those who were insufficiently active had a 23% (OR 0.77, 95% CI 0.75–0.79) reduction of mental health burden and there was a 44% (0.56, 0.54–0.58) reduction in the odds of mental health burden for physically active individuals (Fig. 2 and Supplementary Data 4). Furthermore, the results of subgroup analysis for gender, age, lifestyle behaviors, and chronic diseases in our study were also similar and showed consistency in graded effects (Fig. 2). Moreover, magnitude of the protective role of activity varied from person to person (Fig. 2). Compared with adults aged from 30 to 59 years (0.84, 0.81–0.87), active older people aged 60 years or above had much lower mental health burden (0.68, 0.65–0.71). We also noted that those who followed a healthy lifestyle (no smoking [0.75, 0.72–0.77] or no drinking [0.74, 0.72–0.77]) had lower mental health problems than smokers (0.85, 0.81–0.90) or drinkers (0.90, 0.85–0.96) if they did activity. Additionally, a higher activity level was more closely associated with lower mental health burden for individuals with chronic diseases (0.68, 0.65–0.70) than those without (0.78, 0.75–0.81).

### Significant differences across populations in associations between different forms of activity and mental health

**Fig. 3:**
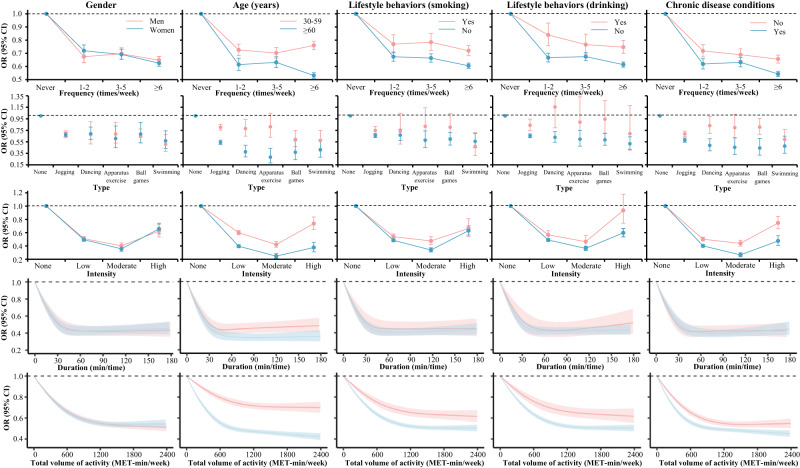
Different forms of leisure time physical activity and mental health in different groups people (*N* = 711,759). Models were adjusted for geographical regions, urbanization, gender, age, marital status, education, employment, income, smoking, alcohol consumption, and year. Chronic diseases must be diagnosed by doctors and defined as having a newly diagnosed chronic condition in the past six months or having a diagnosed chronic condition before six months but suffering from disease symptoms and receiving treatments during the past six months. Bars and ribbons show 95% CIs. OR odds ratio, MET metabolic equivalent of task.

We observed that the associations between different activity formats and mental health varied in different individuals (Fig. 3 and Supplementary Data 5). Both men and women had the lowest mental health burden when they did moderate-intensity swimming six times or above a week for 50 min (Fig. 3). Notably, the appropriate types of activity differed significantly between adults of different ages. For adults aged 30–59 years, playing ball games of moderate intensity for 50 min three or five times a week was associated with lower odds of poor mental health. However, for older adults aged 60 years or above, doing moderate-intensity apparatus exercise for 55 min at least six times a week was strongly associated with lower mental health burden. It is worth noting that older people had a more obvious reduction of mental health burden in all activity features (frequency, duration, types, and intensity) than younger people (Fig. 3). In addition, for individuals with chronic disease, lower odds in mental health burden was observed to be associated with moderate-intensity apparatus exercise for 50 min or more, at least six times a week (Fig. 3). Furthermore, individuals who followed a healthy lifestyle had lower mental health burden compared to those who did not, given the same activity volume (Fig. 3).

## Discussion

This study constitutes the largest study investigating disparate associations between leisure time physical activity features and mental health across different populations to date based on a large-scale nationally representative sample of Chinese individuals. Our findings demonstrated that associations between frequency, duration, type, and intensity of activity and mental health vary in populations across gender, age, lifestyle behaviors, and chronic disease conditions. The dose-response relationship between total activity volume and mental health was first reported, which was highly nonlinear and presented L-shaped with a threshold of 1200 MET-min of activity a week. Notably, merely exercising 3–5 times per week with moderate swimming was significantly associated with lower mental health burden among younger people, while the association was strongly large in older adults aged 60 years or above doing 55-min moderate apparatus exercise at least six times a week. These findings have important implications, suggesting that activity promotion strategies need to be tailored based on population contexts.

Consistent with the results in previous works including randomized controlled trials and prospective cohort studies^9–13^ the association between activity and mental health in our study is plausible after controlling for several confounders. Additionally, the L-shaped dose-response association suggested that most benefits are observed even with low-volume activity, with only minor additional benefits for higher volume. This finding confirmed the curvilinear relation found in physical health gains^18–20^, while the threshold of 1200 MET-week of activity volume found in our study is two times higher than physical health gains required in general guidelines for 600 MET per week^27–29^. Unlike physical fitness, mental health problems are attributed to many complex factors such as biological factors, life experiences, and living environments therefore higher level activity might be more significant related to lower burden^27–31^. There are several potential mechanisms that could explain the protective effect of activity on mental health^32–39^. The physiological mechanisms advocated that activity might decrease mental disorders risk like depression through pathways such as acute neuroendocrine and inflammatory responses to activity and longer-term adaptations^32–34^. Psychosocial and behavioral explanations suggest that activity improves self-efficacy, enhances confidence, and promotes social interactions having impacts on mental health^35,36^. Additionally, environmental factors such as noise, green space, and air pollution also play an important role as a potential moderator of the association between activity and mental health^37–39^. Overall, the L-shaped dose-response association indicated that substantially lower mental health burden was related to even low-level activity, with additional burden decreasing at higher activity levels.

As previous studies mentioned, the above mechanisms may not operate to the same degree across different activity parameters, such as types, frequencies, intensities, and durations, potentially resulting in higher heterogeneity^14,21^. In addition to total volume, it is necessary to explore the relationship for these aspects of activity. Therefore, we further conducted an individual and more precise analysis of specific features and found that activity pattern of six times per week, 50-min duration each time, and moderate intensity is related to the lowest mental health burden. One study found that even if the duration was as short as 15 min, 3 times a week was significantly associated with a lower risk of depressive symptoms^40^. The other study advocated that doing activity for 45 min for three to five times per week was linked to the lowest risk of poor mental health and indicated negative psychological effects from high levels of activity^17^. However, most previous works investigated only one or a few dimensions of activity, which might lead to unreliable or inconsistent results^17,40^. Our results captured the most comprehensive activity aspects so far, suggesting that the optimal activity pattern was higher in duration and frequency, and no adverse effects on mental health were found. Additionally, our results indicated activity of both social and non-social forms such as swimming, ball games, and dancing were associated with reduced mental health burden. Social form activities like ball games, mostly team-based, can enhance communication with people, minimize social withdrawal, decrease feelings of isolation, and promote resilience to stress, which can contribute an additional benefit for mental health^17,41,42^. However, other types of activity can generate equal benefits such as swimming, and dancing. These kinds of activity can greatly reduce feelings of anger, confusion, tension, and depression, and substantially improve self-efficacy^43–45^. Importantly, activity assessed by different parameters can be captured to discriminate different dimensions and offer targeting information for improving activity to reduce mental health burden.

Through specific subgroup analyses, our results suggest that reasonable activity was more strongly related to mental health in older adults, individuals with chronic diseases, and people following a healthy lifestyle (no drinking, no smoking). Older people may experience many stressors such as physical problems, bereavement, and a drop in socioeconomic status with retirement, which can result in isolation, loneliness or psychological distress^46,47^. Individuals with chronic diseases may face reduced mobility, chronic pain, frailty or other health problems, for which they require some form of long-term care^48^. Both older people and individuals with chronic diseases are vulnerable people and may face higher mental health risks^46–48^, making activity particularly important for these populations. For older people, we observed that doing 55-min moderate apparatus exercise at least six times a week has a strongly large association with lower mental health burden. Given the major challenge of population aging^49,50^, providing public spaces such as parks and squares in urban planning and adequate sports facilities in community design could be effective measures to encourage older people to be active and reduce feelings of social isolation and depression. Additionally, our study revealed that dancing and apparatus exercise are more strongly associated with reduced mental health problems among people with chronic diseases. The fact that activity is more correlated with mental health for healthy people might be due to that individuals following a healthy lifestyle can achieve more significant health benefits when doing activity or that mental health problems are less occurring among healthy people. Overall, our study provides robust and comprehensive evidence to support targeting public health policies to reduce mental health burden by promoting physical activity and maximizing positive effects for different people.

As advocated in WHO global guidelines, the concepts of “active living”, “active travel”, and “active workplace” are of great importance^51^. Developing strategies to promote physical activity and reduce mental health burden requires a collective effort, both at national and local levels, involving different disciplines and sectors to implement policies appropriate to a country’s cultural and social environment. Considering people with varying gender, age, lifestyles, and health status, specific programs are needed to create more activity opportunities in different settings, including workplaces, schools, communities, and families targeting various groups. Specifically, reducing barriers for women participating in various sports by improving access to safe equipment and welcoming facilities, enhancing school-sport programs to support adolescents to spend their free time actively, developing workplace policies to encourage adults to be physically active during the work day, improving community-based recreation facilities to promote access for older people doing exercise as well as enhancing their functional capacity, and encouraging healthcare providers support patients to be regularly active may be effective approaches.

## Limitations

This study also had several limitations. First, our risk estimates fundamentally only show correlations because this study is based on cross-sectional surveys, and the causal association between LTPA and mental health cannot be determined. Therefore, alternative study designs including randomized controlled trials or longitudinal design with long follow-up, are needed to determine causality. Second, our study relies on self-reported activity which might be overestimated. However, the assessment of activity using self-reported measures is considered acceptable in large studies due to low-resource settings. Third, participant-reported mental health burden could not effectively identify specific mental health problems, such as stress, depression, or anxiety. This suggests the need for studies to explore the precise association between actual physical activity and specific mental health symptoms by using automatic activity data collection equipment (e.g., wearable sensor) and structured interviews or standardized rating scales.

## Conclusion

Based on large-scale data, we provided essential evidence of the association between LTPA and mental health burden, which can support public health policies for better mental health. Our findings reveal complexities of the disparate linkages between mental health and activity, in the aspects of dose-response nonlinear relationships, diverse set of activity features, and inter-individual differences. Policies targeted at improving physical activity participation are a low-cost approach to reducing mental health burden, while importantly, policies and efforts may need to be optimized for specific subpopulations.

### Supplementary information

Supplementary Information

Description of Additional Supplementary Files

Supplementary Data 1

Supplementary Data 2

Supplementary Data 3

Supplementary Data 4

Supplementary Data 5

Supplementary Data 6

Reporting Summary

## Data Availability

Data tenure was controlled by the Centre for Health Statistics Information, National Health Commission of the People’s Republic of China. The source data for Figs. 1, 2, 3, and Supplementary Fig. 3 can be found in Supplementary Data 3, 4, 5, and 2 respectively. Raw data are not publicly available due to privacy considerations. Data access can be requested by email to the corresponding author.

## Code availability

All codes that support the findings of this study are available online as Supplementary Data 6.
