# Supplementary material for: Large-scale data reveal disparate associations between leisure time physical activity patterns and mental health
Source: Commun Med (Lond). 2023 Dec 21;3:175. doi: 10.1038/s43856-023-00399-2 (PMC10739930; doi:10.1038/s43856-023-00399-2)
Supplement: Supplementary file 1 — Supplementary Information [file 43856_2023_399_MOESM1_ESM.pdf]

Large-scale data reveal disparate associations between leisure time physical activity patterns and mental health

Supplementary material content

Supplementary Method. Study design and dataset description

Supplementary Figure 1. The geographic distribution of sampled counties in NHSS 2003, 2008, 2013, and 2018

Supplementary Figure 2. Study profile

Supplementary Figure 3. Percentage of total population and subgroups with mental health burden for different levels of leisure time physical activity

Supplementary Table 1. Characteristics of leisure time physical activity features of participants

Supplementary Table 2. Characteristics of the participants

Supplementary References.

Supplementary Method. Study design and dataset description

Our study based on the latest four rounds (2003, 2008, 2013 and 2018) of the National Health Services Survey (NHSS) covering all 31 provinces, autonomous regions, and municipalities in the mainland of China. The NHSS has been organized by the National Health Commission (NHC) of the People's Republic of China every fifth year since 1993. It uses multistage stratified cluster sampling and the methods have been described in detail elsewhere.[1,2] Specifically, the mainland of China was divided into east, central, and west regions and then sampled counties from each region stratified by urban and rural areas. To cover 0.02% of the population of the whole country and take into account a 2% non-respondent rate, at least 90 counties and 600 households for each county were needed to be sampled. In stage one, 95 of a possible 2861 counties were randomly selected in 2003, and 94 of which were chosen again in the 2008 survey (one county was excluded because its administrative division had changed). To better represent the increased urbanisation of China, another 52 counties from urban areas and 10 counties from rural areas were randomly selected in addition to those counties involved in the 2008 survey (a total of 156 counties sampled) in 2013 and 2018. In stage two, five streets in urban areas or townships in rural areas from each county were selected at random. In stage three, two urban communities from each urban street or rural villages from each rural township were sampled respectively. In stage four, 60 households were randomly sampled in each administrative community or village (each of which would typically contain 500–3000 households). All respondents aged 15 years or older in the selected household were eligible in this investigation, and questions about children younger than 15 years were answered by adult family members. The questions about leisure time physical activity were only administered to those who were 15 years or older. The NHSS was approved by the institutional review board of the Chinese National Bureau of Statistics. All respondents provided their oral consent to participate in the survey before the interview. In addition to demographic and socioeconomic data (including self-reported income and expenditure), the interviewers, who are trained medical professionals, also obtained comprehensive information about self-reported health status, health-care utilisation, and behavioural factors, such as smoking and alcohol consumption. Across these four years, data relating to leisure time physical activity were available for 98% of individuals aged 15 years or older We then excluded individuals who were missing response data on self-reported mental health burden over the last month (0.19%), gender (0.01%), smoking (1.45%), alcohol consumption (0.46%), self-reported chronic diseases (0.08%), education level (0.04%), occupation (0.06%), income level (1.46%).

Supplementary Figure 1. The geographic distribution of sampled counties in NHSS 2003, 2008, 2013, and 2018

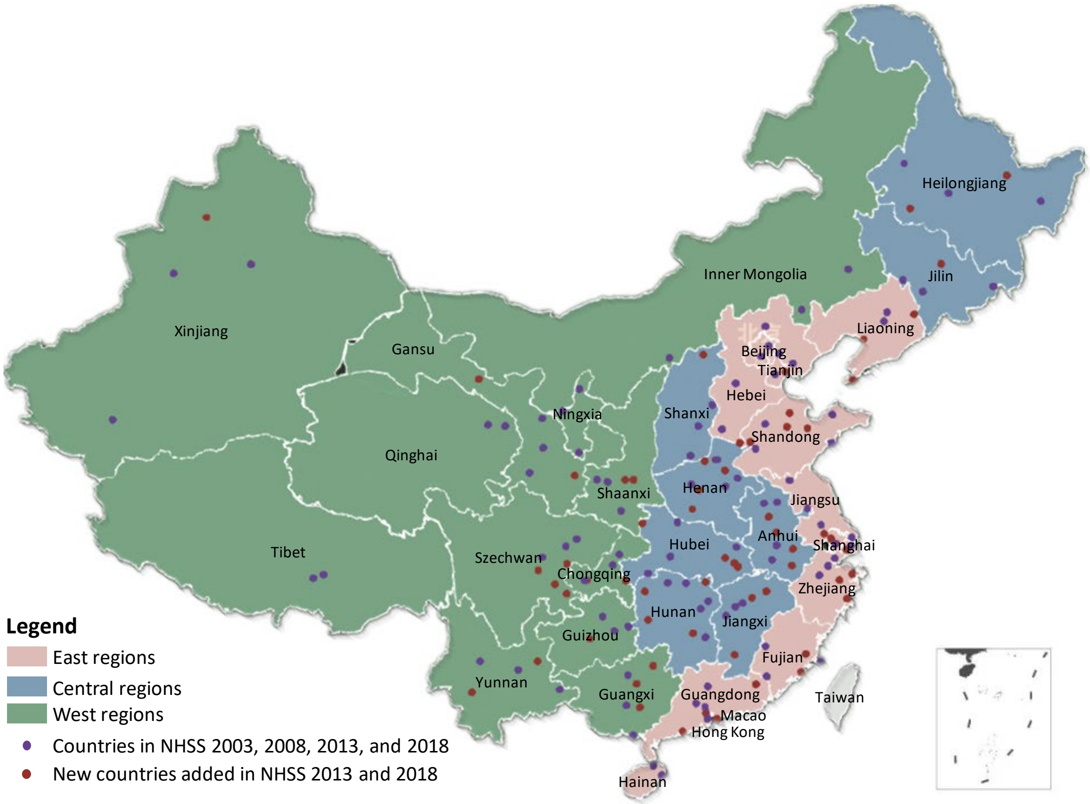

The sampling points selected in the Survey 2003, 2008 ,2013 and 2018 were from three different areas (East, Central, and West) on the basis of geographic location and economic development involving 31 provinces in China. Hong Kong, Macao, and Taiwan were not included in the Survey. One county was selected in the 2003 NHSS but not in 2008, 2013 and 2018 NHSS because of a change of administrative division

Supplementary Figure 2. Study profile

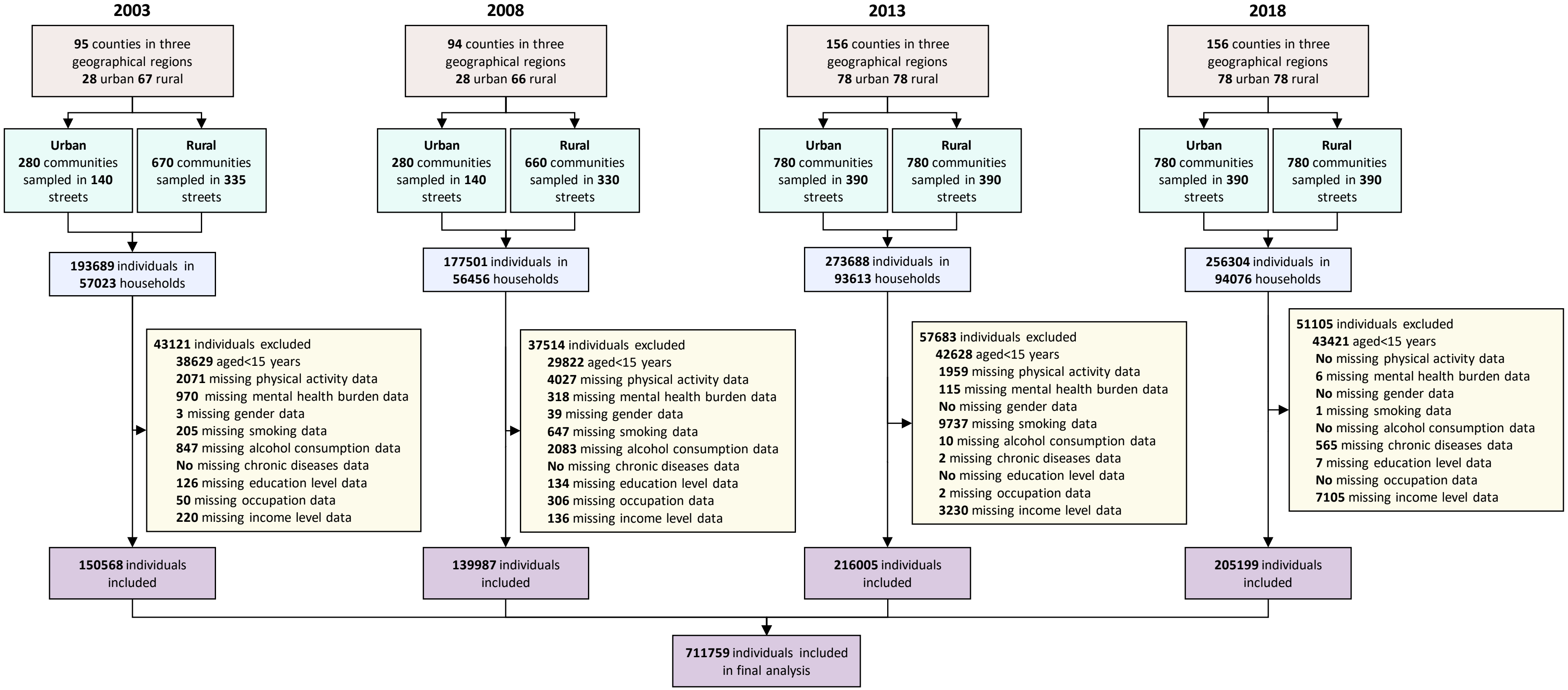

Due to the change of administrative division, one county was selected in 2003 but not in 2008, 2013 and 2018 National Health Service Surveys.

Supplementary Figure 3. Percentage of total population and subgroups with mental health burden for different levels of leisure time physical activity

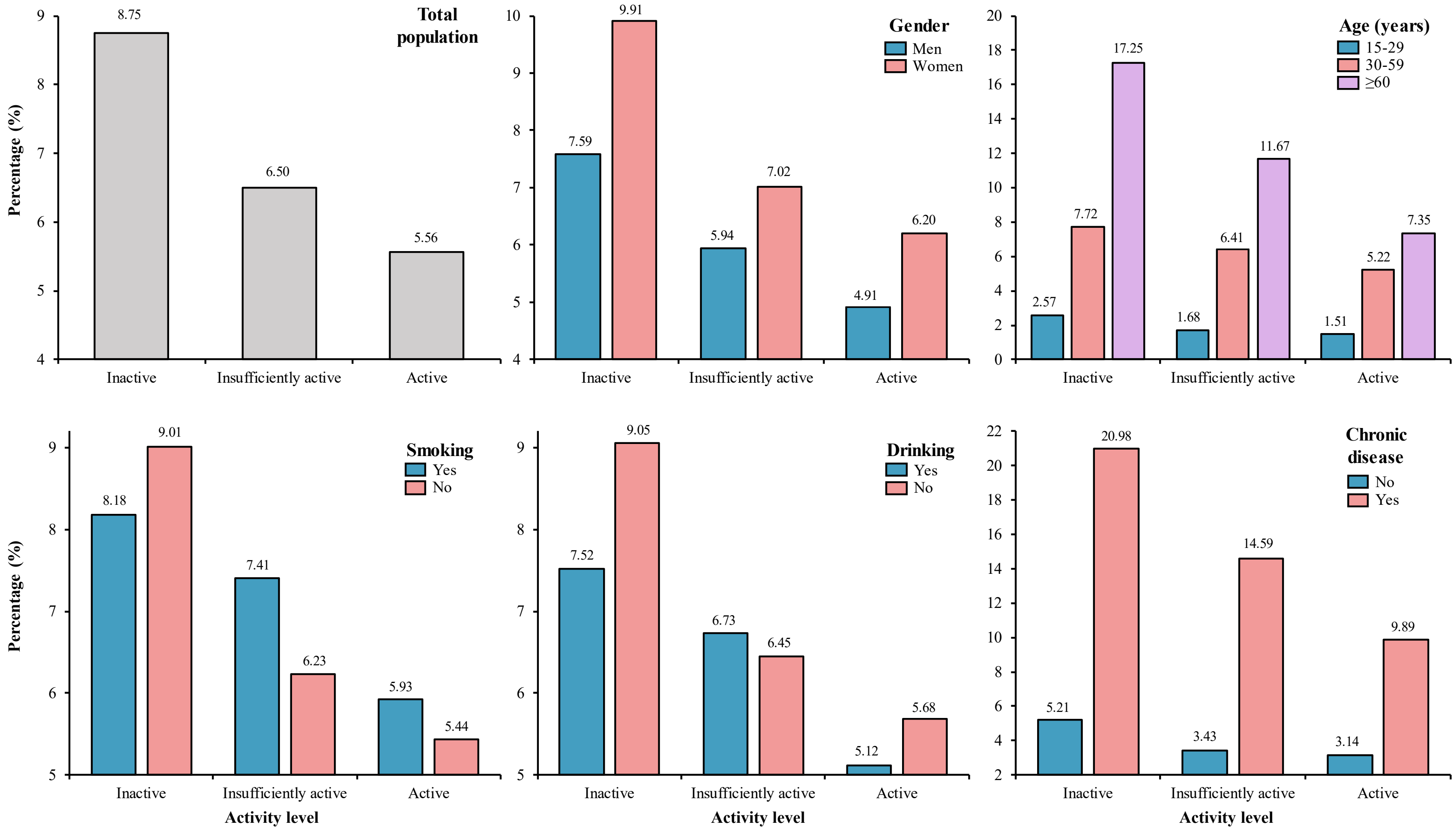

Supplementary Table 1. Characteristics of leisure time physical activity features of participants

	Total	Gender		Age (years)		
		Male	Female	15-29	30-59	≥60
**Individuals**	737172	351853	359906	137819	401928	172012
**Frequency (times/week)**						
Never	502857 (70.7)	250317 (71.1)	252540 (70.2)	97289 (70.6)	299513 (74.5)	106055 (61.7)
1-2	44078 (6.2)	21538 (6.1)	22540 (6.3)	11916 (8.7)	24467 (6.1)	7695 (4.5)
3-5	51645 (7.3)	24938 (7.1)	26707 (7.4)	13495 (9.8)	26724 (6.7)	11426 (6.6)
≥6	113179 (15.9)	55060 (15.7)	58119 (16.2)	15119 (11.0)	51224 (12.7)	46836 (27.2)
**Duration (min/time)**						
0-29	25979 (3.7)	12943 (3.7)	13036 (3.6)	7779 (5.6)	10947 (2.7)	7253 (4.2)
30-59	91314 (12.8)	44102 (12.5)	47212 (13.1)	21731 (15.8)	45573 (11.3)	24010 (14.0)
60-89	75093 (10.6)	35629 (10.1)	39464 (11.0)	10004 (7.3)	39507 (9.8)	25582 (14.9)
90-119	7309 (1.0)	3630 (1.0)	3679 (1.0)	795 (0.6)	3655 (0.9)	2859 (1.7)
120-149	15631 (2.2)	8222 (2.3)	7409 (2.1)	1930 (1.4)	7292 (1.8)	6409 (3.7)
150-179	505 (0.1)	261 (0.1)	244 (0.1)	69 (0.1)	225 (0.1)	211 (0.1)
≥180	2620 (0.4)	1545 (0.4)	1075 (0.3)	314 (0.2)	1115 (0.3)	1191 (0.7)
**Type**						
None	235445 (33.1)	116855 (33.2)	118590 (33.0)	54125 (39.3)	143600 (35.7)	37720 (21.9)
Jogging	37065 (5.2)	18062 (5.1)	19003 (5.3)	6055 (4.4)	18635 (4.6)	12375 (7.2)
Dancing	4665 (0.7)	1296 (0.4)	3369 (0.9)	2302 (1.7)	1630 (0.4)	733 (0.4)
Apparatus exercise	2019 (0.3)	1209 (0.3)	810 (0.2)	671 (0.5)	1006 (0.3)	342 (0.2)
Ball games	7931 (1.1)	5837 (1.7)	2094 (0.6)	5765 (4.2)	1869 (0.5)	297 (0.2)
Swimming	3009 (0.4)	1482 (0.4)	1527 (0.4)	1607 (1.2)	1065 (0.3)	337 (0.2)
**Intensity**						
None	235445 (33.1)	116855 (33.2)	118590 (33.0)	54125 (39.3)	143600 (35.7)	37720 (21.9)
Low	79560 (11.2)	37680 (10.7)	41880 (11.6)	11717 (8.5)	40034 (10.0)	27809 (16.2)
Moderate	27782 (3.9)	16470 (4.7)	11312 (3.1)	11016 (8.0)	12311 (3.1)	4455 (2.6)
High	10478 (1.5)	4597 (1.3)	5881 (1.6)	4901 (3.6)	4083 (1.0)	1494 (0.9)

Notes:

Values are number of participants (N) and proportion (%).

Data of activity type were available in 2003 and 2008, data of activity intensity were available in 2013, and missing data in 2018 using data from 2003, 2008, and 2013.

Supplementary Table 2. Characteristics of the participants

	Overall	Mental health burden		Leisure time physical activity		
		No (%)	Yes (%)	Inactive (%)	Insufficiently Active (%)	Active (%)
**Individuals**	711759	655384 (92.1)	56375 (7.9)	491539 (69.1)	118245 (16.6)	101975 (14.3)
**Gender**						
Men	351853	327447 (93.1)	24406 (6.9)	244584 (69.5)	57011 (16.2)	50258 (14.3)
Women	359906	327937 (91.1)	31969 (8.9)	246955 (68.6)	61234 (17.0)	51717 (14.4)
**Age**						
15-29	137819	134680 (97.7)	3139 (2.3)	94577 (68.6)	30652 (22.2)	12590 (9.1)
30-59	401928	372929 (92.8)	28999 (7.2)	292780 (72.8)	58062 (14.5)	51086 (12.7)
≥60	172012	147775 (85.9)	24237 (14.1)	104182 (60.6)	29531 (17.2)	38299 (22.3)
**Smoking**						
Yes	204164	188248 (92.2)	15916 (7.8)	151603 (74.3)	26895 (13.2)	25666 (12.6)
No	507595	467136 (92.0)	40459 (8.0)	339936 (67.0)	91350 (18.0)	76309 (15.0)
**Alcohol consumption**						
Yes	137843	128139 (93.0)	9704 (7.0)	96024 (69.7)	20990 (15.2)	20829 (15.1)
No	573916	527245 (91.9)	46671 (8.1)	395515 (68.9)	97255 (17.0)	81146 (14.1)
**Chronic diseases**						
No	532219	507375 (95.3)	24844 (4.7)	381141 (71.6)	85747 (16.1)	65331 (12.3)
Yes	179540	148009 (82.4)	31531 (17.6)	110398 (61.5)	32498 (18.1)	36644 (20.4)
**Education level**						
Primary school or below	280677	245710 (87.5)	34967 (12.5)	228692 (81.5)	28486 (10.2)	23499 (8.4)
Middle school	241451	227896 (94.4)	13555 (5.6)	173810 (72.0)	36729 (15.2)	30912 (12.8)
High school	121275	115597 (95.3)	5678 (4.7)	62836 (51.8)	31938 (26.3)	26501 (21.9)
College or above	68356	66181 (96.8)	2175 (3.2)	26201 (38.3)	21092 (30.9)	21063 (30.8)
**Occupation**						
Employed	468175	438032 (93.6)	30143 (6.4)	370200 (79.1)	57175 (12.2)	40800 (8.7)
Retired	85305	77474 (90.8)	7831 (9.2)	27053 (31.7)	20217 (23.7)	38035 (44.6)
Student	36455	36025 (98.8)	430 (1.2)	13546 (37.2)	18000 (49.4)	4909 (13.5)
Unemployed	121824	103853 (85.3)	17971 (14.8)	80740 (66.3)	22853 (18.8)	18231 (15.0)
**Income level**						
Low	187257	166164 (88.7)	21093 (11.3)	147849 (79.0)	23022 (12.3)	16386 (8.8)
Middle	355926	330380 (92.8)	25546 (7.2)	247394 (69.5)	59028 (16.6)	49504 (13.9)
High	168576	158840 (94.2)	9736 (5.8)	96296 (57.1)	36195 (21.5)	36085 (21.4)

Notes:

Values are number of participants (N) and proportion (%).

Low income: the last 25% of the population's annual per capital income in the sampled county at the survey year.

Middle income: Between 25% and 75% of the population's annual per capital income in the sampled county at the survey year.

High income: the top 25% of the population's annual per capital income in the sampled county at the survey year.

Alcohol consumption: have a drink containing alcohol in the past 12 months.

Smoking: have smoked a total of at least 100 cigarettes and has not quit smoking.

Supplementary References

1. Meng Q, Xu L, Zhang Y, et al. Trends in access to health services and financial protection in China between 2003 and 2011: a cross-sectional study. Lancet 2012; 379: 805–14. doi:10.1016/S0140-6736(12)60278-5

2. Wang M, Luo X, Xu S, et al. Trends in smoking prevalence and implication for chronic diseases in China: serial national cross-sectional surveys from 2003 to 2013. Lancet Respir Me. 2019; 7(1): 35-45. doi: 10.1016/S2213-2600(18)30432-6
