# Supplementary material for: Large-scale data reveal disparate associations between leisure time physical activity patterns and mental health
Source: Commun Med (Lond). 2023 Dec 21;3:175. doi: 10.1038/s43856-023-00399-2 (PMC10739930; doi:10.1038/s43856-023-00399-2)
Supplement: Supplementary file 2 — Description of Additional Supplementary Files [file 43856_2023_399_MOESM2_ESM.docx]

**Description of Additional Supplementary Files**

**File name:** Supplementary Data 1

**Description:** The related information extracted from the whole questionnaire of NHSS in our survey.

**File name:** Supplementary Data 2

**Description:** Percentage of total population and subgroups with mental health burden for different levels of leisure time physical activity.

**File name:** Supplementary Data 3

**Description:** Source data of odds ratio values used to generate associations between different forms of leisure time physical activity with mental health in total population.

**File name:** Supplementary Data 4

**Description:** Source data of odds ratio values and exact p values used to generate associations between leisure time physical activity with mental health in different people.

**File name:** Supplementary Data 5

**Description:** Source data of odds ratio values used to generate associations between different forms of leisure time physical activity with mental health in different groups people.

**File name:** Supplementary Data 6

**Description:** R codes for the manuscript “Large-scale data reveal disparate associations between leisure time physical activity patterns and mental health”
