# Supplementary material for: Large-scale data reveal disparate associations between leisure time physical activity patterns and mental health
Source: Commun Med (Lond). 2023 Dec 21;3:175. doi: 10.1038/s43856-023-00399-2 (PMC10739930; doi:10.1038/s43856-023-00399-2)
Supplement: Supplementary file 3 — Supplementary Data 1 [file 43856_2023_399_MOESM3_ESM.docx]

**The related information extracted from the whole questionnaire of NHSS in our survey**

| **No.** | **Question** | | | | | | **Answer** |
| --- | --- | --- | --- | --- | --- | --- | --- |
| Family information | | | | | | | |
| 1 | How many people have lived in your home in the past 6 months? (Including relatives and friends, nannies, etc. who have lived for over 6 months) | | | | | |  |
| 12 | What was the total annual income of your family in the previous year? (yuan) | | | | | |  |
| Demographic information of each household member | | | | | | | |
| Household members coding (01, the householder; Other members, according to the order of investigation) | | 01 | 02 | 03 | 04 | 05 | … |
| 1 | Name of household member: (01, name of the householder) |  |  |  |  |  |  |
| 2 | Relationship with the householder: (1) Householder (2) Spouse (3) Child (4) Grandchild  (5) Parent (6) Grandparent (7) Brother or sister (8) Others |  |  |  |  |  |  |
| 3 | Gender: (1) Male (2) Female |  |  |  |  |  |  |
| 4 | Date of birth: (Year/ Month) |  |  |  |  |  |  |
| 5 | Marital status: (1) Unmarried (2) Married (3) Divorce (4) Widowed (5) Others |  |  |  |  |  |  |
| 6 | Level of education:  (1) None (2) Primary school (3) Junior middle school (4) Senior high school/technical school  (5) Secondary specialized school (6) Junior college (7) University and above |  |  |  |  |  |  |
| 7 | Employment status: (1) Employed (2) Retired (3) Student (4) Unemployed |  |  |  |  |  |  |
| Chronic diseases information of each household member | | | | | | | |
| 1 | Have you suffered from any chronic diseases diagnosed by doctors? (1) Yes; (2) No |  |  |  |  |  |  |
|  | (if more than one disease, please fill in the name of disease according to the severity of disease from severe to mild) |  |  |  |  |  |  |
| 2 | (1) Name of the first disease: |  |  |  |  |  |  |
| 3 | The code of the first disease: |  |  |  |  |  |  |
| 4 | When were you diagnosed with it? (1) Over 6 months ago; (2) Within the past 6 months |  |  |  |  |  |  |
| 5 | Have you been treated within the past 6 months? (1) Yes; (2) No |  |  |  |  |  |  |
| 6 | (2) Name of the second disease: |  |  |  |  |  |  |
| 7 | The code of the second disease: |  |  |  |  |  |  |
| 8 | When were you diagnosed with it? (1) Over 6 months ago; (2) Within the past 6 months |  |  |  |  |  |  |
| 9 | Have you been treated within the past 6 months? (1) Yes; (2) No |  |  |  |  |  |  |
| 10 | (3) Name of the third disease: |  |  |  |  |  |  |
| 11 | The code of the third disease: |  |  |  |  |  |  |
| 12 | When were you diagnosed with it? (1) Over 6 months ago; (2) Within the past 6 months |  |  |  |  |  |  |
| 13 | Have you been treated within the past 6 months? (1) Yes; (2) No |  |  |  |  |  |  |
| Health status and related lifestyle behaviors of each household member | | | | | | | |
| 1 | Do you smoke?  (1) Yes (have smoked a total of at least 100 cigarettes); (2) Have quit smoking; (3) Never |  |  |  |  |  |  |
| 16 | Have you had a drink containing alcohol in the past 12 months? (1) Yes; (2) No |  |  |  |  |  |  |
| 19 | In the past 6 months, how many times per week do you do physical exercise on average?  (1) 6 or more; (2) 3 to 5; (3) 1 to 2; (4) less than 1; (5) Never |  |  |  |  |  |  |
| 20 | What type of exercise do you participate in most frequently?  (1) Ball games (2) Apparatus exercise (3) Dancing (4) Swimming (5) Trotting or jogging, tai chi, yoga (6) Others |  |  |  |  |  |  |
| 21 | How long do you do physical exercise on average each time? (minutes) |  |  |  |  |  |  |
| 22 | What is the average intensity of physical exercise each time? (the feeling of self-breathing and rapid heartbeat)  (1) Light (2) Moderate (3) Vigorous |  |  |  |  |  |  |
| 25 | Now thinking about your mental health, which includes stress, depression, anxiety, and problems with emotions, whether you have these problems during the past 30 days was your mental health not good?  (1) No problems (2) Moderate problems (3) Severe problems |  |  |  |  |  |  |

**Notes:**

Chronic diseases must be diagnosed by doctors and defined as having a newly diagnosed chronic condition in the past six months or having a diagnosed chronic condition before six months but suffering from diseases symptoms and receiving treatments during the past six months.

Smoking is defined as one has smoked a total of at least 100 cigarettes and has not quit smoking.

Alcohol consumption is defined as one has had a drink containing alcohol in the past 12 months.

There are minor adjustments of the questionnaire in different surveys. Data of activity type were available in 2003 and 2008, data of activity intensity were available in 2013, and missing data in 2018 using data from 2003, 2008, and 2013.
