# Supplementary material for: Large-scale data reveal disparate associations between leisure time physical activity patterns and mental health
Source: Commun Med (Lond). 2023 Dec 21;3:175. doi: 10.1038/s43856-023-00399-2 (PMC10739930; doi:10.1038/s43856-023-00399-2)
Supplement: Supplementary file 8 — Supplementary Data 6 [file 43856_2023_399_MOESM8_ESM.docx]

**Large-scale data reveal disparate associations between** **leisure time physical activity patterns and mental health**

**#Figure 1**

library(ggplot2)

load("C:/Users/hustbim/Desktop/data1.RData")

factor<-data1$factor

or<-data1$or

lor<-data1$lor

uor<-data1$uor

**#Frequency**

a<-ggplot(data1,aes(factor,or))+

geom_hline(aes(yintercept=1), colour="black", linetype="dashed")+

geom_errorbar(aes(ymin=lor, ymax=uor), width=.1,size=0.8)+

geom_pointrange(aes(factor,or,ymin=lor, ymax=uor),fill='red', color='black', shape=21, lwd=0.9,size=5)+

geom_line(aes(factor,or),group = 1,color="red",size=1)

a+theme (panel.background = element_rect(fill = NA),

panel.grid.major.y = element_blank(),panel.grid.minor.y = element_blank(),

panel.grid.major.x = element_blank(),panel.grid.minor.x = element_blank(),

axis.line = element_line(size = 1, colour = "black"),

axis.text = element_text(size=10,colour = "black",family="A"),

axis.ticks = element_line(size = 1),

axis.ticks.length.y = unit(.15, "cm"),

axis.ticks.length.x = unit(.15, "cm"),

title=element_text(size=12, color="black"), legend.position = "none")+

scale_y_continuous(limits = c(0.6,1), expand = c(0.05,0),breaks = seq(0.6,1,0.1))+

scale_x_discrete(expand = c(0.05,0) , labels = c("Never","1-2","3-5","≥6"))+

labs (y="OR (95% CI)",x="Frequency (times/week)")

**#Type**

b<-ggplot(data1,aes(factor,or))+

geom_hline(aes(yintercept=1), colour="black", linetype="dashed")+

geom_errorbar(aes(ymin=lor, ymax=uor), width=.1,size=0.8)+

geom_pointrange(aes(factor,or,ymin=lor, ymax=uor),fill='red', color='black', shape=21, lwd=0.9,size=5)+

geom_line(aes(factor,or),group = 1,color="red",size=1)

b+theme (panel.background = element_rect(fill = NA),

panel.grid.major.y = element_blank(),panel.grid.minor.y = element_blank(),

panel.grid.major.x = element_blank(),panel.grid.minor.x = element_blank(),

axis.line = element_line(size = 1, colour = "black"),

axis.text = element_text(size=10,colour = "black",family="A"),

axis.ticks = element_line(size = 1),

axis.ticks.length.y = unit(.15, "cm"),

axis.ticks.length.x = unit(.15, "cm"),

title=element_text(size=12, color="black"), legend.position = "none")+

scale_y_continuous(limits = c(0.4,1), expand = c(0.05,0),breaks = seq(0.4,1,0.1))+

scale_x_discrete(expand = c(0.05,0) , labels = c("None","Jogging","Dancing","Apparatua exercise","Ball games","Swimming"))+

labs (y="OR (95% CI)",x="Type")

**#Intensity**

c<-ggplot(data1,aes(factor,or))+

geom_hline(aes(yintercept=1), colour="black", linetype="dashed")+

geom_errorbar(aes(ymin=lor, ymax=uor), width=.1,size=0.8)+

geom_pointrange(aes(factor,or,ymin=lor, ymax=uor),fill='red', color='black', shape=21, lwd=0.9,size=5)+

geom_line(aes(factor,or),group = 1,color="red",size=1)

c+theme (panel.background = element_rect(fill = NA),

panel.grid.major.y = element_blank(),panel.grid.minor.y = element_blank(),

panel.grid.major.x = element_blank(),panel.grid.minor.x = element_blank(),

axis.line = element_line(size = 1, colour = "black"),

axis.text = element_text(size=10,colour = "black",family="A"),

axis.ticks = element_line(size = 1),

axis.ticks.length.y = unit(.15, "cm"),

axis.ticks.length.x = unit(.15, "cm"),

title=element_text(size=12, color="black"), legend.position = "none")+

scale_y_continuous(limits = c(0.3,1), expand = c(0.05,0),breaks = seq(0.3,1,0.1))+

scale_x_discrete(expand = c(0.05,0) , labels = c("Inactive","Low","Moderate","High"))+

labs (y="OR (95% CI)",x="Intensity")

library(ggplot2)

library(rms)

library(Hmisc)

load("C:/Users/hustbim/Desktop/data2.RData")

depress22<-data2$depress2

ltpa2<-data2$ltpa1

urb_rur12<-data2$urb_rur

area12<-data2$area

marriage22<-data2$ marriage2

edu32<-data2$edu2

smoif22<-data2$smoif2

drifre22<-data2$drifre2

work12<-data2$work1

income42<-data$2income2

age2<-data2$age

gender2<-data2$gender

spotim2<-data2$spotim

year2<-data2$year

datab<-data.frame(depress22,ltpa2, urb_rur12, area12, marriage22, edu32, smoif22, drifre22, income42, age2, gender2,work12,spotim2,year2)

dd2 <- datadist(datab)

options(datadist='dd2')

**#Duration**

fit3 <- lrm(depress22~ rcs(spotim2,4)+urb_rur12+area12 +marriage22+edu32+smoif22+drifre22+income42+age2+ gender2+work12+year2, data=datab)

print(fit3)

dd2$limits$spotim2[2]<-0

fit3=update(fit3)

OR3<-Predict(fit3, spotim2,fun=exp,ref.zero = TRUE)

p5<-ggplot(OR3)

anova(fit3)

d<-ggplot()+geom_line(data=OR3, aes(spotim2,yhat),linetype="solid",size=1,alpha = 0.5,colour="red")+

geom_hline(yintercept=1, linetype=2,size=0.6)+

geom_hline(yintercept=0.42, linetype=2,size=0.6)+

geom_vline(xintercept=45, linetype=2,size=0.6)+

geom_ribbon(data=OR3, aes(spotim2,ymin = lower, ymax = upper),alpha = 0.15,fill="red")

d+theme (panel.background = element_rect(fill = NA),

panel.grid.major.y = element_blank(),panel.grid.minor.y = element_blank(),

panel.grid.major.x = element_blank(),panel.grid.minor.x = element_blank(),

axis.line = element_line(size = 1, colour = "black"),

axis.text = element_text(size=10,colour = "black",family="A"),

axis.ticks = element_line(size = 1),

axis.ticks.length.y = unit(.15, "cm"),

axis.ticks.length.x = unit(.15, "cm"),

title=element_text(size=12, color="black"), legend.position = "none")+

scale_y_continuous(limits = c(0.3,1), expand = c(0.05,0),breaks = seq(0.3,1,0.1))+

scale_x_continuous(limits = c(0,180), expand = c(0.05,0),breaks = seq(0,180,30))+

labs( x="Duration (min/time)", y="OR (95%CI)")

**#Total volume**

fit2 <- lrm(depress22~ rcs(ltpa2,3)+urb_rur12+area12 +marriage22+edu32+smoif22+drifre22+income42+age2+gender2+work12+ year2, data=datab)

print(fit2)

dd2$limits$ltpa2[2]<-0

fit2=update(fit2)

OR2<-Predict(fit2, ltpa2,fun=exp,ref.zero = TRUE)

p3<-ggplot(OR2)

anova(fit2)

e<-ggplot()+geom_line(data=OR2, aes(ltpa2,yhat),linetype="solid",size=1,alpha = 0.5,colour="red")+

geom_ribbon(data=OR2, aes(ltpa2,ymin = lower, ymax = upper),alpha = 0.15,fill="red")

e+geom_hline(yintercept=1, linetype=2,size=0.6)+

geom_hline(yintercept=0.53, linetype=2,size=0.6)+

geom_vline(xintercept=1200, linetype=2,size=0.6)+

theme (panel.background = element_rect(fill = NA),

panel.grid.major.y = element_blank(),panel.grid.minor.y = element_blank(),

panel.grid.major.x = element_blank(),panel.grid.minor.x = element_blank(),

axis.line = element_line(size = 1, colour = "black"),

axis.text = element_text(size=10,colour = "black",family="A"),

axis.ticks = element_line(size = 1),

axis.ticks.length.y = unit(.15, "cm"),

axis.ticks.length.x = unit(.15, "cm"),

title=element_text(size=12, color="black"), legend.position = "none")+

scale_y_continuous(limits = c(0.4,1.1), expand = c(0.05,0),breaks = seq(0.4,1.1,0.1))+

scale_x_continuous(limits = c(0,2400), expand = c(0.05,0),breaks = seq(0,2400,300))+

labs( x="Total volume of physical activity (MET-min/week)", y="OR (95%CI)")

**#Figure 2**

library(forestplot)

load("C:/Users/hustbim/Desktop/data.RData")

windowsFonts(A = windowsFont("Times New Roman"))

labeltext <- as.matrix(data[,1:6])

coef <- with(data, cbind(or1, or2))

low <- with(data, cbind(lci1, lci2))

high <- with(data, cbind(uci1, uci2))

forestplot(labeltext,

mean = coef,

lower = low ,

upper = high,

is.summary=c(T,F,F,F,F,T,F,F,F,F,F,F,T,F,F,F,F,T,F,F,F,F,T,F,F,F,F,T,T),

graph.pos = 5,graphwidth = unit(80,'mm'),

txt_gp=fpTxtGp(ticks=gpar(cex=0.8),summary=gpar(cex=0.8),cex=0.8,label=gpar(fontfamily="A")),

boxsize = 0.45,

lineheight = unit(5.5,'mm'),

line.margin =unit(4.5,'mm'),

colgap = unit(2,'mm'),

zero = 1,

lwd.zero = 1,

lwd.ci = 1,

grid = structure(c(1.0), gp = gpar(col = "grey50", lty = 2)),

col=fpColors(box=c("#FF9999","#009FC3"),summary='grey50',lines =c("#FF9999","#009FC3"),zero = 'white'),

lwd.xaxis=1,xticks = c(0.4,0.6,0.8,1,1.2),

xlog=TRUE)

**#Figure 3**

**#Frequency**

library(gridExtra)

library(ggplot2)

windowsFonts(A = windowsFont("Times New Roman"))

load("C:/Users/hustbim/Desktop/zhexian/spo/data1.RData")

factor<-data1$factor

group<-data1$group

or1<-data1$or1

lor1<-data1$lor1

uor1<-data1$uor1

or2<-data1$or2

lor2<-data1$lor2

uor2<-data1$uor2

or3<-data1$or3

lor3<-data1$lor3

uor3<-data1$uor3

or4<-data1$or4

lor4<-data1$lor4

uor4<-data1$uor4

or5<-data1$or5

lor5<-data1$lor5

uor5<-data1$uor5

a<-ggplot(data1,aes(factor,or1,color=gro, group=gro))+

geom_errorbar(aes(ymin=lor1, ymax=uor1), width=.15,size=0.4,position=position_dodge(0))+

geom_hline(aes(yintercept=1), colour="black", linetype="dashed")+

geom_point(size=2,position=position_dodge(0))+

geom_line(size=0.4,position=position_dodge(0))

a1<-a+ theme()+

theme (panel.background = element_rect(fill = NA),

panel.grid.major.y = element_blank(),panel.grid.minor.y = element_blank(),

panel.grid.major.x = element_blank(),panel.grid.minor.x = element_blank(),

axis.line = element_line(size = 1, colour = "black"),

axis.text = element_text(size=6,colour = "black",family="A"),

axis.ticks = element_line(size = 1),

axis.ticks.length.y = unit(.15, "cm"),

axis.ticks.length.x = unit(.15, "cm"),

title=element_text(size=8, color="black"), legend.position = "none")+

scale_y_continuous(limits = c(0.5,1), expand = c(0.05,0),breaks = seq(0.5,1,0.1))+

scale_x_discrete(expand = c(0.05,0) , labels = c("Never","1-2","3-5","≥6"))+

scale_color_manual(values=c("#FF9999","#009FC3"))+

labs (y="OR (95% CI)",x="Frequency (times/week)")

a2=a1+guides(fill=F)

b<-ggplot(data1,aes(factor,or2,color=gro, group=gro))+

geom_errorbar(aes(ymin=lor2, ymax=uor2), width=.15,size=0.4,position=position_dodge(0))+

geom_hline(aes(yintercept=1), colour="black", linetype="dashed")+

geom_point(size=2,position=position_dodge(0))+

geom_line(size=0.4,position=position_dodge(0))

b1<-b+ theme(axis.title.y=element_blank(),axis.text.y = element_blank())+

theme (panel.background = element_rect(fill = NA),

panel.grid.major.y = element_blank(),panel.grid.minor.y = element_blank(),

panel.grid.major.x = element_blank(),panel.grid.minor.x = element_blank(),

axis.line = element_line(size = 1, colour = "black"),

axis.text = element_text(size=6,colour = "black",family="A"),

axis.ticks = element_line(size = 1),

axis.ticks.length.y = unit(.15, "cm"),

axis.ticks.length.x = unit(.15, "cm"),

title=element_text(size=8, color="black"), legend.position = "none")+

scale_y_continuous(limits = c(0.5,1), expand = c(0.05,0),breaks = seq(0.5,1,0.1))+

scale_x_discrete(expand = c(0.05,0) , labels = c("Never","1-2","3-5","≥6"))+

scale_color_manual(values=c("#FF9999","#009FC3"))+

labs (y="OR (95% CI)",x="Frequency (times/week)")

b2=b1+guides(fill=F)

c<-ggplot(data1,aes(factor,or3,color=gro, group=gro))+

geom_errorbar(aes(ymin=lor3, ymax=uor3), width=.15,size=0.4,position=position_dodge(0))+

geom_hline(aes(yintercept=1), colour="black", linetype="dashed")+

geom_point(size=2,position=position_dodge(0))+

geom_line(size=0.4,position=position_dodge(0))

c1<-c+ theme(axis.title.y=element_blank(),axis.text.y = element_blank())+

theme (panel.background = element_rect(fill = NA),

panel.grid.major.y = element_blank(),panel.grid.minor.y = element_blank(),

panel.grid.major.x = element_blank(),panel.grid.minor.x = element_blank(),

axis.line = element_line(size = 1, colour = "black"),

axis.text = element_text(size=6,colour = "black",family="A"),

axis.ticks = element_line(size = 1),

axis.ticks.length.y = unit(.15, "cm"),

axis.ticks.length.x = unit(.15, "cm"),

title=element_text(size=8, color="black"), legend.position = "none")+

scale_y_continuous(limits = c(0.5,1), expand = c(0.05,0),breaks = seq(0.5,1,0.1))+

scale_x_discrete(expand = c(0.05,0) , labels = c("Never","1-2","3-5","≥6"))+

scale_color_manual(values=c("#FF9999","#009FC3"))+

labs (y="OR (95% CI)",x="Frequency (times/week)")

c2=c1+guides(fill=F)

d<-ggplot(data1,aes(factor,or4,color=gro, group=gro))+

geom_errorbar(aes(ymin=lor4, ymax=uor4), width=.15,size=0.4,position=position_dodge(0))+

geom_hline(aes(yintercept=1), colour="black", linetype="dashed")+

geom_point(size=2,position=position_dodge(0))+

geom_line(size=0.4,position=position_dodge(0))

d1<-d+ theme(axis.title.y=element_blank(),axis.text.y = element_blank())+

theme (panel.background = element_rect(fill = NA),

panel.grid.major.y = element_blank(),panel.grid.minor.y = element_blank(),

panel.grid.major.x = element_blank(),panel.grid.minor.x = element_blank(),

axis.line = element_line(size = 1, colour = "black"),

axis.text = element_text(size=6,colour = "black",family="A"),

axis.ticks = element_line(size = 1),

axis.ticks.length.y = unit(.15, "cm"),

axis.ticks.length.x = unit(.15, "cm"),

title=element_text(size=8, color="black"), legend.position = "none")+

scale_y_continuous(limits = c(0.5,1), expand = c(0.05,0),breaks = seq(0.5,1,0.1))+

scale_x_discrete(expand = c(0.05,0) , labels = c("Never","1-2","3-5","≥6"))+

scale_color_manual(values=c("#FF9999","#009FC3"))+

labs (y="OR (95% CI)",x="Frequency (times/week)")

d2=d1+guides(fill=F)

e<-ggplot(data1,aes(factor,or5,color=gro, group=gro))+

geom_errorbar(aes(ymin=lor5, ymax=uor5), width=.15,size=0.4,position=position_dodge(0))+

geom_hline(aes(yintercept=1), colour="black", linetype="dashed")+

geom_point(size=2,position=position_dodge(0))+

geom_line(size=0.4,position=position_dodge(0))

e1<-e+ theme(axis.title.y=element_blank(),axis.text.y = element_blank())+

theme (panel.background = element_rect(fill = NA),

panel.grid.major.y = element_blank(),panel.grid.minor.y = element_blank(),

panel.grid.major.x = element_blank(),panel.grid.minor.x = element_blank(),

axis.line = element_line(size = 1, colour = "black"),

axis.text = element_text(size=6,colour = "black",family="A"),

axis.ticks = element_line(size = 1),

axis.ticks.length.y = unit(.15, "cm"),

axis.ticks.length.x = unit(.15, "cm"),

title=element_text(size=8, color="black"), legend.position = "none")+

scale_y_continuous(limits = c(0.5,1), expand = c(0.05,0),breaks = seq(0.5,1,0.1))+

scale_x_discrete(expand = c(0.05,0) , labels = c("Never","1-2","3-5","≥6"))+

scale_color_manual(values=c("#FF9999","#009FC3"))+

labs (y="OR (95% CI)",x="Frequency (times/week)")

e2=e1+guides(fill=F)

grid.arrange(a2,b2,c2,d2,e2, ncol=5, nrow =1)

**# Type**

load("C:/Users/hustbim/Desktop/zhexian/spo/data2.RData")

factor<-data2$factor

group<-data2$group

or1<-data2$or1

lor1<-data2$lor1

uor1<-data2$uor1

or2<-data2$or2

lor2<-data2$lor2

uor2<-data2$uor2

or3<-data2$or3

lor3<-data2$lor3

uor3<-data2$uor3

or4<-data2$or4

lor4<-data2$lor4

uor4<-data2$uor4

or5<-data2$or5

lor5<-data2$lor5

uor5<-data2$uor5

aa<-ggplot(data2,aes(factor,or1,color=gro, group=gro))+

geom_errorbar(aes(ymin=lor1, ymax=uor1), width=.25,size=0.4,position=position_dodge(0))+

geom_hline(aes(yintercept=1), colour="black", linetype="dashed")+

geom_point(size=2,position=position_dodge(0))+

geom_line(size=0.4,position=position_dodge(0))

aa1<-aa+ theme()+

theme (panel.background = element_rect(fill = NA),

panel.grid.major.y = element_blank(),panel.grid.minor.y = element_blank(),

panel.grid.major.x = element_blank(),panel.grid.minor.x = element_blank(),

axis.line = element_line(size = 1, colour = "black"),

axis.text = element_text(size=6,colour = "black",family="A"),

axis.ticks = element_line(size = 1),

axis.ticks.length.y = unit(.15, "cm"),

axis.ticks.length.x = unit(.15, "cm"),

title=element_text(size=8, color="black"), legend.position = "none")+

scale_y_continuous(limits = c(0.15,1.75), expand = c(0.05,0),breaks = seq(0.15,1.75,0.2))+

scale_x_discrete(expand = c(0.05,0) , labels = c("None","Jogging","Dancing","Apparatua","Ball","Swimming"))+

scale_color_manual(values=c("#FF9999","#009FC3"))+

labs (y="OR (95% CI)",x="Type")

aa2=aa1+guides(fill=F)

bb<-ggplot(data2,aes(factor,or2,color=gro, group=gro))+

geom_errorbar(aes(ymin=lor2, ymax=uor2), width=.25,size=0.4,position=position_dodge(0))+

geom_hline(aes(yintercept=1), colour="black", linetype="dashed")+

geom_point(size=2,position=position_dodge(0))+

geom_line(size=0.4,position=position_dodge(0))

bb1<-bb+ theme(axis.title.y=element_blank(),axis.text.y = element_blank())+

theme (panel.background = element_rect(fill = NA),

panel.grid.major.y = element_blank(),panel.grid.minor.y = element_blank(),

panel.grid.major.x = element_blank(),panel.grid.minor.x = element_blank(),

axis.line = element_line(size = 1, colour = "black"),

axis.text = element_text(size=6,colour = "black",family="A"),

axis.ticks = element_line(size = 1),

axis.ticks.length.y = unit(.15, "cm"),

axis.ticks.length.x = unit(.15, "cm"),

title=element_text(size=8, color="black"), legend.position = "none")+

scale_y_continuous(limits = c(0.15,1.75), expand = c(0.05,0),breaks = seq(0.15,1.75,0.2))+

scale_x_discrete(expand = c(0.05,0) , labels = c("None","Jogging","Dancing","Apparatua","Ball","Swimming"))+

scale_color_manual(values=c("#FF9999","#009FC3"))+

labs (y="OR (95% CI)",x="Type")

bb2=bb1+guides(fill=F)

cc<-ggplot(data2,aes(factor,or3,color=gro, group=gro))+

geom_errorbar(aes(ymin=lor3, ymax=uor3), width=.25,size=0.4,position=position_dodge(0))+

geom_hline(aes(yintercept=1), colour="black", linetype="dashed")+

geom_point(size=2,position=position_dodge(0))+

geom_line(size=0.4,position=position_dodge(0))

cc1<-cc+ theme(axis.title.y=element_blank(),axis.text.y = element_blank())+

theme (panel.background = element_rect(fill = NA),

panel.grid.major.y = element_blank(),panel.grid.minor.y = element_blank(),

panel.grid.major.x = element_blank(),panel.grid.minor.x = element_blank(),

axis.line = element_line(size = 1, colour = "black"),

axis.text = element_text(size=6,colour = "black",family="A"),

axis.ticks = element_line(size = 1),

axis.ticks.length.y = unit(.15, "cm"),

axis.ticks.length.x = unit(.15, "cm"),

title=element_text(size=8, color="black"), legend.position = "none")+

scale_y_continuous(limits = c(0.15,1.75), expand = c(0.05,0),breaks = seq(0.15,1.75,0.2))+

scale_x_discrete(expand = c(0.05,0) , labels = c("None","Jogging","Dancing","Apparatua","Ball","Swimming"))+

scale_color_manual(values=c("#FF9999","#009FC3"))+

labs (y="OR (95% CI)",x="Type")

cc2=cc1+guides(fill=F)

dd<-ggplot(data2,aes(factor,or4,color=gro, group=gro))+

geom_errorbar(aes(ymin=lor4, ymax=uor4), width=.25,size=0.4,position=position_dodge(0))+

geom_hline(aes(yintercept=1), colour="black", linetype="dashed")+

geom_point(size=2,position=position_dodge(0))+

geom_line(size=0.4,position=position_dodge(0))

dd1<-dd+ theme(axis.title.y=element_blank(),axis.text.y = element_blank())+

theme (panel.background = element_rect(fill = NA),

panel.grid.major.y = element_blank(),panel.grid.minor.y = element_blank(),

panel.grid.major.x = element_blank(),panel.grid.minor.x = element_blank(),

axis.line = element_line(size = 1, colour = "black"),

axis.text = element_text(size=6,colour = "black",family="A"),

axis.ticks = element_line(size = 1),

axis.ticks.length.y = unit(.15, "cm"),

axis.ticks.length.x = unit(.15, "cm"),

title=element_text(size=8, color="black"), legend.position = "none")+

scale_y_continuous(limits = c(0.15,1.75), expand = c(0.05,0),breaks = seq(0.15,1.75,0.2))+

scale_x_discrete(expand = c(0.05,0) , labels = c("None","Jogging","Dancing","Apparatua","Ball","Swimming"))+

scale_color_manual(values=c("#FF9999","#009FC3"))+

labs (y="OR (95% CI)",x="Type")

dd2=dd1+guides(fill=F)

ee<-ggplot(data2,aes(factor,or5,color=gro, group=gro))+

geom_errorbar(aes(ymin=lor5, ymax=uor5), width=.25,size=0.4,position=position_dodge(0))+

geom_hline(aes(yintercept=1), colour="black", linetype="dashed")+

geom_point(size=2,position=position_dodge(0))+

geom_line(size=0.4,position=position_dodge(0))

ee1<-ee+ theme(axis.title.y=element_blank(),axis.text.y = element_blank())+

theme (panel.background = element_rect(fill = NA),

panel.grid.major.y = element_blank(),panel.grid.minor.y = element_blank(),

panel.grid.major.x = element_blank(),panel.grid.minor.x = element_blank(),

axis.line = element_line(size = 1, colour = "black"),

axis.text = element_text(size=6,colour = "black",family="A"),

axis.ticks = element_line(size = 1),

axis.ticks.length.y = unit(.15, "cm"),

axis.ticks.length.x = unit(.15, "cm"),

title=element_text(size=8, color="black"), legend.position = "none")+

scale_y_continuous(limits = c(0.15,1.75), expand = c(0.05,0),breaks = seq(0.15,1.75,0.2))+

scale_x_discrete(expand = c(0.05,0) , labels = c("None","Jogging","Dancing","Apparatua","Ball","Swimming"))+

scale_color_manual(values=c("#FF9999","#009FC3"))+

labs (y="OR (95% CI)",x="Type)")

ee2=ee1+guides(fill=F)

grid.arrange(aa2,bb2,cc2,dd2,ee2, ncol=5, nrow =1)

**# Intensity**

load("C:/Users/hustbim/Desktop/zhexian/spo/data3.RData")

factor<-data3$factor

group<-data3$group

or1<-data3$or1

lor1<-data3$lor1

uor1<-data3$uor1

or2<-data3$or2

lor2<-data3$lor2

uor2<-data3$uor2

or3<-data3$or3

lor3<-data3$lor3

uor3<-data3$uor3

or4<-data3$or4

lor4<-data3$lor4

uor4<-data3$uor4

or5<-data3$or5

lor5<-data3$lor5

uor5<-data3$uor5

aaa<-ggplot(data3,aes(factor,or1,color=gro, group=gro))+

geom_errorbar(aes(ymin=lor1, ymax=uor1), width=.15,size=0.4,position=position_dodge(0))+

geom_hline(aes(yintercept=1), colour="black", linetype="dashed")+

geom_point(size=2,position=position_dodge(0))+

geom_line(size=0.4,position=position_dodge(0))

aaa1<-aaa+ theme()+

theme (panel.background = element_rect(fill = NA),

panel.grid.major.y = element_blank(),panel.grid.minor.y = element_blank(),

panel.grid.major.x = element_blank(),panel.grid.minor.x = element_blank(),

axis.line = element_line(size = 1, colour = "black"),

axis.text = element_text(size=6,colour = "black",family="A"),

axis.ticks = element_line(size = 1),

axis.ticks.length.y = unit(.15, "cm"),

axis.ticks.length.x = unit(.15, "cm"),

title=element_text(size=8, color="black"), legend.position = "none")+

scale_y_continuous(limits = c(0.2,1.2), expand = c(0.05,0),breaks = seq(0.2,1.2,0.2))+

scale_x_discrete(expand = c(0.05,0) , labels = c("Inactive","Low","Moderate","High"))+

scale_color_manual(values=c("#FF9999","#009FC3"))+

labs (y="OR (95% CI)",x="Intensity")

aaa2=aaa1+guides(fill=F)

bbb<-ggplot(data3,aes(factor,or2,color=gro, group=gro))+

geom_errorbar(aes(ymin=lor2, ymax=uor2), width=.15,size=0.4,position=position_dodge(0))+

geom_hline(aes(yintercept=1), colour="black", linetype="dashed")+

geom_point(size=2,position=position_dodge(0))+

geom_line(size=0.4,position=position_dodge(0))

bbb1<-bbb+ theme(axis.title.y=element_blank(),axis.text.y = element_blank())+

theme (panel.background = element_rect(fill = NA),

panel.grid.major.y = element_blank(),panel.grid.minor.y = element_blank(),

panel.grid.major.x = element_blank(),panel.grid.minor.x = element_blank(),

axis.line = element_line(size = 1, colour = "black"),

axis.text = element_text(size=6,colour = "black",family="A"),

axis.ticks = element_line(size = 1),

axis.ticks.length.y = unit(.15, "cm"),

axis.ticks.length.x = unit(.15, "cm"),

title=element_text(size=8, color="black"), legend.position = "none")+

scale_y_continuous(limits = c(0.2,1.2), expand = c(0.05,0),breaks = seq(0.2,1.2,0.2))+

scale_x_discrete(expand = c(0.05,0) , labels = c("Inactive","Low","Moderate","High"))+

scale_color_manual(values=c("#FF9999","#009FC3"))+

labs (y="OR (95% CI)",x="Intensity")

bbb2=bbb1+guides(fill=F)

ccc<-ggplot(data3,aes(factor,or3,color=gro, group=gro))+

geom_errorbar(aes(ymin=lor3, ymax=uor3), width=.15,size=0.4,position=position_dodge(0))+

geom_hline(aes(yintercept=1), colour="black", linetype="dashed")+

geom_point(size=2,position=position_dodge(0))+

geom_line(size=0.4,position=position_dodge(0))

ccc1<-ccc+ theme(axis.title.y=element_blank(),axis.text.y = element_blank())+

theme (panel.background = element_rect(fill = NA),

panel.grid.major.y = element_blank(),panel.grid.minor.y = element_blank(),

panel.grid.major.x = element_blank(),panel.grid.minor.x = element_blank(),

axis.line = element_line(size = 1, colour = "black"),

axis.text = element_text(size=6,colour = "black",family="A"),

axis.ticks = element_line(size = 1),

axis.ticks.length.y = unit(.15, "cm"),

axis.ticks.length.x = unit(.15, "cm"),

title=element_text(size=8, color="black"), legend.position = "none")+

scale_y_continuous(limits = c(0.2,1.2), expand = c(0.05,0),breaks = seq(0.2,1.2,0.2))+

scale_x_discrete(expand = c(0.05,0) , labels = c("Inactive","Low","Moderate","High"))+

scale_color_manual(values=c("#FF9999","#009FC3"))+

labs (y="OR (95% CI)",x="Intensity")

ccc2=ccc1+guides(fill=F)

ddd<-ggplot(data3,aes(factor,or4,color=gro, group=gro))+

geom_errorbar(aes(ymin=lor4, ymax=uor4), width=.15,size=0.4,position=position_dodge(0))+

geom_hline(aes(yintercept=1), colour="black", linetype="dashed")+

geom_point(size=2,position=position_dodge(0))+

geom_line(size=0.4,position=position_dodge(0))

ddd1<-ddd+ theme(axis.title.y=element_blank(),axis.text.y = element_blank())+

theme (panel.background = element_rect(fill = NA),

panel.grid.major.y = element_blank(),panel.grid.minor.y = element_blank(),

panel.grid.major.x = element_blank(),panel.grid.minor.x = element_blank(),

axis.line = element_line(size = 1, colour = "black"),

axis.text = element_text(size=6,colour = "black",family="A"),

axis.ticks = element_line(size = 1),

axis.ticks.length.y = unit(.15, "cm"),

axis.ticks.length.x = unit(.15, "cm"),

title=element_text(size=8, color="black"), legend.position = "none")+

scale_y_continuous(limits = c(0.2,1.2), expand = c(0.05,0),breaks = seq(0.2,1.2,0.2))+

scale_x_discrete(expand = c(0.05,0) , labels = c("Inactive","Low","Moderate","High"))+

scale_color_manual(values=c("#FF9999","#009FC3"))+

labs (y="OR (95% CI)",x="Intensity")

ddd2=ddd1+guides(fill=F)

eee<-ggplot(data3,aes(factor,or5,color=gro, group=gro))+

geom_errorbar(aes(ymin=lor5, ymax=uor5), width=.15,size=0.4,position=position_dodge(0))+

geom_hline(aes(yintercept=1), colour="black", linetype="dashed")+

geom_point(size=2,position=position_dodge(0))+

geom_line(size=0.4,position=position_dodge(0))

eee1<-eee+ theme(axis.title.y=element_blank(),axis.text.y = element_blank())+

theme (panel.background = element_rect(fill = NA),

panel.grid.major.y = element_blank(),panel.grid.minor.y = element_blank(),

panel.grid.major.x = element_blank(),panel.grid.minor.x = element_blank(),

axis.line = element_line(size = 1, colour = "black"),

axis.text = element_text(size=6,colour = "black",family="A"),

axis.ticks = element_line(size = 1),

axis.ticks.length.y = unit(.15, "cm"),

axis.ticks.length.x = unit(.15, "cm"),

title=element_text(size=8, color="black"), legend.position = "none")+

scale_y_continuous(limits = c(0.2,1.2), expand = c(0.05,0),breaks = seq(0.2,1.2,0.2))+

scale_x_discrete(expand = c(0.05,0) , labels = c("Inactive","Low","Moderate","High"))+

scale_color_manual(values=c("#FF9999","#009FC3"))+

labs (y="OR (95% CI)",x="Intensity")

eee2=eee1+guides(fill=F)

grid.arrange(aaa2,bbb2,ccc2,ddd2,eee2, ncol=5, nrow =1)

**#Duration**

library(ggplot2)

library(rms)

library(Hmisc)

library(gridExtra)

load("C:/Users/hustbim/Desktop/yangtiao/gender1.RData")

depress22<-gender1$depress2

ltpa2<-gender1$ltpa1

urb_rur12<-gender1$urb_rur

area12<-gender1$area

marriage22<-gender1$ marriage2

edu32<-gender1$edu2

smoif22<-gender1$smoif2

drifre22<-gender1$drifre2

work12<-gender1$work1

income42<-gender1$income2

age2<-gender1$age

spotim2<-gender1$spotim

year2<-gender1$year

gender1b<-data.frame(depress22,ltpa2, urb_rur12, area12, marriage22, edu32, smoif22, drifre22, income42, age2,work12,spotim2,year2)

dd1<- datadist(gender1b)

options(datadist='dd1')

fit1 <- lrm(depress22~ rcs(spotim2,4)+urb_rur12+area12 +marriage22+edu32+smoif22+drifre22+income42+age2+work12+year2, data=gender1b)

print(fit1)

dd1$limits$spotim2[2]<-0

fit1=update(fit1)

OR1<-Predict(fit1, spotim2,fun=exp,ref.zero = TRUE)

p1<-ggplot(OR1)

anova(fit1)

load("C:/Users/hustbim/Desktop/yangtiao/gender2.RData")

depress22<-gender2$depress2

ltpa2<-gender2$ltpa1

urb_rur12<-gender2$urb_rur

area12<-gender2$area

marriage22<-gender2$ marriage2

edu32<-gender2$edu2

smoif22<-gender2$smoif2

drifre22<-gender2$drifre2

work12<-gender2$work1

income42<-gender2$income2

age2<-gender2$age

spotim2<-gender2$spotim

year2<-gender2$year

gender2b<-data.frame(depress22,ltpa2, urb_rur12, area12, marriage22, edu32, smoif22, drifre22, income42, age2, work12,spotim2,year2)

dd2<- datadist(gender2b)

options(datadist='dd2')

fit2 <- lrm(depress22~ rcs(spotim2,4)+urb_rur12+area12 +marriage22+edu32+smoif22+drifre22+income42+age2+work12+year2, data=gender2b)

print(fit2)

dd2$limits$spotim2[2]<-0

fit2=update(fit2)

OR2<-Predict(fit2, spotim2,fun=exp,ref.zero = TRUE)

p2<-ggplot(OR2)

anova(fit2)

m<-ggplot()+geom_line(data=OR1, aes(spotim2,yhat),linetype="solid",size=0.6,alpha = 0.3,colour="red")+

geom_ribbon(data=OR1, aes(spotim2,ymin = lower, ymax = upper),alpha = 0.1,fill="red")+

geom_line(data=OR2, aes(spotim2,yhat),linetype="solid",size=0.6,alpha = 0.7,colour="lightblue")+

geom_ribbon(data=OR2, aes(spotim2,ymin = lower, ymax = upper),alpha = 0.5,fill="lightblue")+

geom_hline(yintercept=1, linetype=2,size=0.6)

m1<-m+ theme (panel.background = element_rect(fill = NA),

panel.grid.major.y = element_blank(),panel.grid.minor.y = element_blank(),

panel.grid.major.x = element_blank(),panel.grid.minor.x = element_blank(),

axis.line = element_line(size = 1, colour = "black"),

axis.text = element_text(size=6,colour = "black",family="A"),

axis.ticks = element_line(size = 1),

axis.ticks.length.y = unit(.15, "cm"),

axis.ticks.length.x = unit(.15, "cm"),

title=element_text(size=8, color="black"), legend.position = "none")+

scale_y_continuous(limits = c(0.2,1), expand = c(0.05,0),breaks = seq(0.2,1,0.2))+

scale_x_continuous(limits = c(0,180), expand = c(0.05,0),breaks = seq(0,180,30))+

labs( x="Duration (min/time)", y="OR (95%CI)")

m2=m1+guides(fill=F)

load("C:/Users/hustbim/Desktop/yangtiao/age52.RData")

depress22<-age52$depress2

ltpa2<-age52$ltpa1

urb_rur12<-age52$urb_rur

area12<-age52$area

marriage22<-age52$ marriage2

edu32<-age52$edu2

smoif22<-age52$smoif2

drifre22<-age52$drifre2

work12<-age52$work1

income42<-age52$income2

gender2<-age52$gender

spotim2<-age52$spotim

year2<-age52$year

age52b<-data.frame(depress22,ltpa2, urb_rur12, area12, marriage22, edu32, smoif22, drifre22, income42, gender2,work12,spotim2,year2)

dd1<- datadist(age52b)

options(datadist='dd1')

fit1 <- lrm(depress22~ rcs(spotim2,4)+urb_rur12+area12 +marriage22+edu32+smoif22+drifre22+income42+gender2+work12+year2, data=age52b)

print(fit1)

dd1$limits$spotim2[2]<-0

fit1=update(fit1)

OR1<-Predict(fit1, spotim2,fun=exp,ref.zero = TRUE)

p1<-ggplot(OR1)

anova(fit1)

load("C:/Users/hustbim/Desktop/yangtiao/age53.RData")

depress22<-age53$depress2

ltpa2<-age53$ltpa1

urb_rur12<-age53$urb_rur

area12<-age53$area

marriage22<-age53$ marriage2

edu32<-age53$edu2

smoif22<-age53$smoif2

drifre22<-age53$drifre2

work12<-age53$work1

income42<-age53$income2

gender2<-age53$gender

spotim2<-age53$spotim

year2<-age53$year

age53b<-data.frame(depress22,ltpa2, urb_rur12, area12, marriage22, edu32, smoif22, drifre22, income42, gender2, work12,spotim2,year2)

dd2<- datadist(age53b)

options(datadist='dd2')

fit2 <- lrm(depress22~ rcs(spotim2,4)+urb_rur12+area12 +marriage22+edu32+smoif22+drifre22+income42+gender2+work12+year2, data=age53b)

print(fit2)

dd2$limits$spotim2[2]<-0

fit2=update(fit2)

OR2<-Predict(fit2, spotim2,fun=exp,ref.zero = TRUE)

p2<-ggplot(OR2)

anova(fit2)

r<-ggplot()+geom_line(data=OR1, aes(spotim2,yhat),linetype="solid",size=0.6,alpha = 0.3,colour="red")+

geom_ribbon(data=OR1, aes(spotim2,ymin = lower, ymax = upper),alpha = 0.1,fill="red")+

geom_line(data=OR2, aes(spotim2,yhat),linetype="solid",size=0.6,alpha = 0.7,colour="lightblue")+

geom_ribbon(data=OR2, aes(spotim2,ymin = lower, ymax = upper),alpha = 0.5,fill="lightblue")+

geom_hline(yintercept=1, linetype=2,size=0.6)

r1<-r+ theme(axis.title.y=element_blank(),axis.text.y = element_blank())+

theme (panel.background = element_rect(fill = NA),

panel.grid.major.y = element_blank(),panel.grid.minor.y = element_blank(),

panel.grid.major.x = element_blank(),panel.grid.minor.x = element_blank(),

axis.line = element_line(size = 1, colour = "black"),

axis.text = element_text(size=6,colour = "black",family="A"),

axis.ticks = element_line(size = 1),

axis.ticks.length.y = unit(.15, "cm"),

axis.ticks.length.x = unit(.15, "cm"),

title=element_text(size=8, color="black"), legend.position = "none")+

scale_y_continuous(limits = c(0.2,1), expand = c(0.05,0),breaks = seq(0.2,1,0.2))+

scale_x_continuous(limits = c(0,180), expand = c(0.05,0),breaks = seq(0,180,30))+

labs( x="Duration (min/time)", y="OR (95%CI)")

r2=r1+guides(fill=F)

load("C:/Users/hustbim/Desktop/yangtiao/smoif21.RData")

depress22<-smoif21$depress2

ltpa2<-smoif21$ltpa1

urb_rur12<-smoif21$urb_rur

area12<-smoif21$area

marriage22<-smoif21$ marriage2

edu32<-smoif21$edu2

age2<-smoif21$age

drifre22<-smoif21$drifre2

work12<-smoif21$work1

income42<-smoif21$income2

gender2<-smoif21$gender

spotim2<-smoif21$spotim

year2<-smoif21$year

smoif21b<-data.frame(depress22,ltpa2, urb_rur12, area12, marriage22, edu32, age2, drifre22, income42, gender2,work12,spotim2,year2)

dd1<- datadist(smoif21b)

options(datadist='dd1')

fit1 <- lrm(depress22~ rcs(spotim2,4)+urb_rur12+area12 +marriage22+edu32+age2+drifre22+income42+gender2+work12+year2, data=smoif21b)

print(fit1)

dd1$limits$spotim2[2]<-0

fit1=update(fit1)

OR1<-Predict(fit1, spotim2,fun=exp,ref.zero = TRUE)

p1<-ggplot(OR1)

anova(fit1)

load("C:/Users/hustbim/Desktop/yangtiao/smoif22.RData")

depress22<-smoif22$depress2

ltpa2<-smoif22$ltpa1

urb_rur12<-smoif22$urb_rur

area12<-smoif22$area

marriage22<-smoif22$ marriage2

edu32<-smoif22$edu2

age2<-smoif22$age

drifre22<-smoif22$drifre2

work12<-smoif22$work1

income42<-smoif22$income2

gender2<-smoif22$gender

spotim2<-smoif22$spotim

year2<-smoif22$year

smoif22b<-data.frame(depress22,ltpa2, urb_rur12, area12, marriage22, edu32, age2, drifre22, income42, gender2, work12,spotim2,year2)

dd2<- datadist(smoif22b)

options(datadist='dd2')

fit2 <- lrm(depress22~ rcs(spotim2,4)+urb_rur12+area12 +marriage22+edu32+age2+drifre22+income42+gender2+work12+year2, data=smoif22b)

print(fit2)

dd2$limits$spotim2[2]<-0

fit2=update(fit2)

OR2<-Predict(fit2, spotim2,fun=exp,ref.zero = TRUE)

p2<-ggplot(OR2)

anova(fit2)

s<-ggplot()+geom_line(data=OR1, aes(spotim2,yhat),linetype="solid",size=0.6,alpha = 0.3,colour="red")+

geom_ribbon(data=OR1, aes(spotim2,ymin = lower, ymax = upper),alpha = 0.1,fill="red")+

geom_line(data=OR2, aes(spotim2,yhat),linetype="solid",size=0.6,alpha = 0.7,colour="lightblue")+

geom_ribbon(data=OR2, aes(spotim2,ymin = lower, ymax = upper),alpha = 0.5,fill="lightblue")+

geom_hline(yintercept=1, linetype=2,size=0.6)

s1<-s+ theme(axis.title.y=element_blank(),axis.text.y = element_blank())+

theme (panel.background = element_rect(fill = NA),

panel.grid.major.y = element_blank(),panel.grid.minor.y = element_blank(),

panel.grid.major.x = element_blank(),panel.grid.minor.x = element_blank(),

axis.line = element_line(size = 1, colour = "black"),

axis.text = element_text(size=6,colour = "black",family="A"),

axis.ticks = element_line(size = 1),

axis.ticks.length.y = unit(.15, "cm"),

axis.ticks.length.x = unit(.15, "cm"),

title=element_text(size=8, color="black"), legend.position = "none")+

scale_y_continuous(limits = c(0.2,1), expand = c(0.05,0),breaks = seq(0.2,1,0.2))+

scale_x_continuous(limits = c(0,180), expand = c(0.05,0),breaks = seq(0,180,30))+

labs( x="Duration (min/time)", y="OR (95%CI)")

s2=s1+guides(fill=F)

load("C:/Users/hustbim/Desktop/yangtiao/drifre21.RData")

depress22<-drifre21$depress2

ltpa2<-drifre21$ltpa1

urb_rur12<-drifre21$urb_rur

area12<-drifre21$area

marriage22<-drifre21$ marriage2

edu32<-drifre21$edu2

smoif22<-drifre21$smoif2

age2<-drifre21$age

work12<-drifre21$work1

income42<-drifre21$income2

gender2<-drifre21$gender

spotim2<-drifre21$spotim

year2<-drifre21$year

drifre21b<-data.frame(depress22,ltpa2, urb_rur12, area12, marriage22, edu32, smoif22, age2, income42, gender2,work12,spotim2,year2)

dd1<- datadist(drifre21b)

options(datadist='dd1')

fit1 <- lrm(depress22~ rcs(spotim2,4)+urb_rur12+area12 +marriage22+edu32+smoif22+age2+income42+gender2+work12+year2, data=drifre21b)

print(fit1)

dd1$limits$spotim2[2]<-0

fit1=update(fit1)

OR1<-Predict(fit1, spotim2,fun=exp,ref.zero = TRUE)

p1<-ggplot(OR1)

anova(fit1)

load("C:/Users/hustbim/Desktop/yangtiao/drifre22.RData")

depress22<-drifre22$depress2

ltpa2<-drifre22$ltpa1

urb_rur12<-drifre22$urb_rur

area12<-drifre22$area

marriage22<-drifre22$ marriage2

edu32<-drifre22$edu2

smoif22<-drifre22$smoif2

age2<-drifre22$age

work12<-drifre22$work1

income42<-drifre22$income2

gender2<-drifre22$gender

spotim2<-drifre22$spotim

year2<-drifre22$year

drifre22b<-data.frame(depress22,ltpa2, urb_rur12, area12, marriage22, edu32, smoif22, age2, income42, gender2, work12,spotim2,year2)

dd2<- datadist(drifre22b)

options(datadist='dd2')

fit2 <- lrm(depress22~ rcs(spotim2,4)+urb_rur12+area12 +marriage22+edu32+smoif22+age2+income42+gender2+work12+year2, data=drifre22b)

print(fit2)

dd2$limits$spotim2[2]<-0

fit2=update(fit2)

OR2<-Predict(fit2, spotim2,fun=exp,ref.zero = TRUE)

p2<-ggplot(OR2)

anova(fit2)

t<-ggplot()+geom_line(data=OR1, aes(spotim2,yhat),linetype="solid",size=0.6,alpha = 0.3,colour="red")+

geom_ribbon(data=OR1, aes(spotim2,ymin = lower, ymax = upper),alpha = 0.1,fill="red")+

geom_line(data=OR2, aes(spotim2,yhat),linetype="solid",size=0.6,alpha = 0.7,colour="lightblue")+

geom_ribbon(data=OR2, aes(spotim2,ymin = lower, ymax = upper),alpha = 0.5,fill="lightblue")+

geom_hline(yintercept=1, linetype=2,size=0.6)

t1<-t+ theme(axis.title.y=element_blank(),axis.text.y = element_blank())+

theme (panel.background = element_rect(fill = NA),

panel.grid.major.y = element_blank(),panel.grid.minor.y = element_blank(),

panel.grid.major.x = element_blank(),panel.grid.minor.x = element_blank(),

axis.line = element_line(size = 1, colour = "black"),

axis.text = element_text(size=6,colour = "black",family="A"),

axis.ticks = element_line(size = 1),

axis.ticks.length.y = unit(.15, "cm"),

axis.ticks.length.x = unit(.15, "cm"),

title=element_text(size=8, color="black"), legend.position = "none")+

scale_y_continuous(limits = c(0.2,1), expand = c(0.05,0),breaks = seq(0.2,1,0.2))+

scale_x_continuous(limits = c(0,180), expand = c(0.05,0),breaks = seq(0,180,30))+

labs( x="Duration (min/time)", y="OR (95%CI)")

t2=t1+guides(fill=F)

load("C:/Users/hustbim/Desktop/yangtiao/manbing10.RData")

depress22<-manbing10$depress2

ltpa2<-manbing10$ltpa1

urb_rur12<-manbing10$urb_rur

area12<-manbing10$area

marriage22<-manbing10$ marriage2

edu32<-manbing10$edu2

smoif22<-manbing10$smoif2

drifre22<-manbing10$drifre2

work12<-manbing10$work1

income42<-manbing10$income2

gender2<-manbing10$gender

spotim2<-manbing10$spotim

age2<-manbing10$age

year2<-manbing10$year

manbing10b<-data.frame(depress22,ltpa2, urb_rur12, area12, marriage22, edu32, smoif22, drifre22, income42, gender2,work12,spotim2,age2,year2)

dd1<- datadist(manbing10b)

options(datadist='dd1')

fit1 <- lrm(depress22~ rcs(spotim2,4)+urb_rur12+area12 +marriage22+edu32+smoif22+drifre22+income42+gender2+work12+age2+year2, data=manbing10b)

print(fit1)

dd1$limits$spotim2[2]<-0

fit1=update(fit1)

OR1<-Predict(fit1, spotim2,fun=exp,ref.zero = TRUE)

p1<-ggplot(OR1)

anova(fit1)

load("C:/Users/hustbim/Desktop/yangtiao/manbing11.RData")

depress22<-manbing11$depress2

ltpa2<-manbing11$ltpa1

urb_rur12<-manbing11$urb_rur

area12<-manbing11$area

marriage22<-manbing11$ marriage2

edu32<-manbing11$edu2

smoif22<-manbing11$smoif2

drifre22<-manbing11$drifre2

work12<-manbing11$work1

income42<-manbing11$income2

gender2<-manbing11$gender

spotim2<-manbing11$spotim

age2<-manbing11$age

year2<-manbing11$year

manbing11b<-data.frame(depress22,ltpa2, urb_rur12, area12, marriage22, edu32, smoif22, drifre22, income42, gender2, work12,spotim2,age2,year2)

dd2<- datadist(manbing11b)

options(datadist='dd2')

fit2 <- lrm(depress22~ rcs(spotim2,4)+urb_rur12+area12 +marriage22+edu32+smoif22+drifre22+income42+gender2+work12+age2+year2, data=manbing11b)

print(fit2)

dd2$limits$spotim2[2]<-0

fit2=update(fit2)

OR2<-Predict(fit2, spotim2,fun=exp,ref.zero = TRUE)

p2<-ggplot(OR2)

anova(fit2)

u<-ggplot()+geom_line(data=OR1, aes(spotim2,yhat),linetype="solid",size=0.6,alpha = 0.3,colour="red")+

geom_ribbon(data=OR1, aes(spotim2,ymin = lower, ymax = upper),alpha = 0.1,fill="red")+

geom_line(data=OR2, aes(spotim2,yhat),linetype="solid",size=0.6,alpha = 0.7,colour="lightblue")+

geom_ribbon(data=OR2, aes(spotim2,ymin = lower, ymax = upper),alpha = 0.5,fill="lightblue")+

geom_hline(yintercept=1, linetype=2,size=0.6)

u1<-u+ theme(axis.title.y=element_blank(),axis.text.y = element_blank())+

theme (panel.background = element_rect(fill = NA),

panel.grid.major.y = element_blank(),panel.grid.minor.y = element_blank(),

panel.grid.major.x = element_blank(),panel.grid.minor.x = element_blank(),

axis.line = element_line(size = 1, colour = "black"),

axis.text = element_text(size=6,colour = "black",family="A"),

axis.ticks = element_line(size = 1),

axis.ticks.length.y = unit(.15, "cm"),

axis.ticks.length.x = unit(.15, "cm"),

title=element_text(size=8, color="black"), legend.position = "none")+

scale_y_continuous(limits = c(0.2,1), expand = c(0.05,0),breaks = seq(0.2,1,0.2))+

scale_x_continuous(limits = c(0,180), expand = c(0.05,0),breaks = seq(0,180,30))+

labs( x="Duration (min/time)", y="OR (95%CI)")

u2=u1+guides(fill=F)

grid.arrange(m2,r2,s2,t2,u2, ncol=5, nrow =1)

**#Total volume**

library(ggplot2)

library(rms)

library(Hmisc)

library(gridExtra)

load("C:/Users/hustbim/Desktop/yangtiao/gender1.RData")

depress22<-gender1$depress2

ltpa2<-gender1$ltpa1

urb_rur12<-gender1$urb_rur

area12<-gender1$area

marriage22<-gender1$ marriage2

edu32<-gender1$edu2

smoif22<-gender1$smoif2

drifre22<-gender1$drifre2

work12<-gender1$work1

income42<-gender1$income2

age2<-gender1$age

spotim2<-gender1$spotim

year2<-gender1$year

gender1b<-data.frame(depress22,ltpa2, urb_rur12, area12, marriage22, edu32, smoif22, drifre22, income42, age2,work12,spotim2,year2)

dd1<- datadist(gender1b)

options(datadist='dd1')

fit1 <- lrm(depress22~ rcs(ltpa2,3)+urb_rur12+area12 +marriage22+edu32+smoif22+drifre22+income42+age2+work12+year2, data=gender1b)

print(fit1)

dd1$limits$spotim2[2]<-0

fit1=update(fit1)

OR1<-Predict(fit1, ltpa2,fun=exp,ref.zero = TRUE)

p1<-ggplot(OR1)

anova(fit1)

load("C:/Users/hustbim/Desktop/yangtiao/gender2.RData")

depress22<-gender2$depress2

ltpa2<-gender2$ltpa1

urb_rur12<-gender2$urb_rur

area12<-gender2$area

marriage22<-gender2$ marriage2

edu32<-gender2$edu2

smoif22<-gender2$smoif2

drifre22<-gender2$drifre2

work12<-gender2$work1

income42<-gender2$income2

age2<-gender2$age

spotim2<-gender2$spotim

year2<-gender2$year

gender2b<-data.frame(depress22,ltpa2, urb_rur12, area12, marriage22, edu32, smoif22, drifre22, income42, age2, work12,spotim2,year2)

dd2<- datadist(gender2b)

options(datadist='dd2')

fit2 <- lrm(depress22~ rcs(ltpa2,3)+urb_rur12+area12 +marriage22+edu32+smoif22+drifre22+income42+age2+work12+year2, data=gender2b)

print(fit2)

dd2$limits$spotim2[2]<-0

fit2=update(fit2)

OR2<-Predict(fit2, ltpa2,fun=exp,ref.zero = TRUE)

p2<-ggplot(OR2)

anova(fit2)

mm<-ggplot()+geom_line(data=OR1, aes(ltpa2,yhat),linetype="solid",size=0.6,alpha = 0.3,colour="red")+

geom_ribbon(data=OR1, aes(ltpa2,ymin = lower, ymax = upper),alpha = 0.1,fill="red")+

geom_line(data=OR2, aes(ltpa2,yhat),linetype="solid",size=0.6,alpha = 0.7,colour="lightblue")+

geom_ribbon(data=OR2, aes(ltpa2,ymin = lower, ymax = upper),alpha = 0.5,fill="lightblue")+

geom_hline(yintercept=1, linetype=2,size=0.6)

mm1<-mm+ theme(panel.background = element_rect(fill = NA),

panel.grid.major.y = element_blank(),panel.grid.minor.y = element_blank(),

panel.grid.major.x = element_blank(),panel.grid.minor.x = element_blank(),

axis.line = element_line(size = 1, colour = "black"),

axis.text = element_text(size=6,colour = "black",family="A"),

axis.ticks = element_line(size = 1),

axis.ticks.length.y = unit(.15, "cm"),

axis.ticks.length.x = unit(.15, "cm"),

title=element_text(size=8, color="black"), legend.position = "none")+

scale_y_continuous(limits = c(0.35,1.0), expand = c(0.05,0),breaks = seq(0.4,1.0,0.2))+

scale_x_continuous(limits = c(0,2400), expand = c(0.05,0),breaks = seq(0,2400,600))+

labs( x="Total volume (MET-min/week)", y="OR (95%CI)")

mm2=mm1+guides(fill=F)

load("C:/Users/hustbim/Desktop/yangtiao/age52.RData")

depress22<-age52$depress2

ltpa2<-age52$ltpa1

urb_rur12<-age52$urb_rur

area12<-age52$area

marriage22<-age52$ marriage2

edu32<-age52$edu2

smoif22<-age52$smoif2

drifre22<-age52$drifre2

work12<-age52$work1

income42<-age52$income2

gender2<-age52$gender

spotim2<-age52$spotim

year2<-age52$year

age52b<-data.frame(depress22,ltpa2, urb_rur12, area12, marriage22, edu32, smoif22, drifre22, income42, gender2,work12,spotim2,year2)

dd1<- datadist(age52b)

options(datadist='dd1')

fit1 <- lrm(depress22~ rcs(ltpa2,3)+urb_rur12+area12 +marriage22+edu32+smoif22+drifre22+income42+gender2+work12+year2, data=age52b)

print(fit1)

dd1$limits$spotim2[2]<-0

fit1=update(fit1)

OR1<-Predict(fit1, ltpa2,fun=exp,ref.zero = TRUE)

p1<-ggplot(OR1)

anova(fit1)

load("C:/Users/hustbim/Desktop/yangtiao/age53.RData")

depress22<-age53$depress2

ltpa2<-age53$ltpa1

urb_rur12<-age53$urb_rur

area12<-age53$area

marriage22<-age53$ marriage2

edu32<-age53$edu2

smoif22<-age53$smoif2

drifre22<-age53$drifre2

work12<-age53$work1

income42<-age53$income2

gender2<-age53$gender

spotim2<-age53$spotim

year2<-age53$year

age53b<-data.frame(depress22,ltpa2, urb_rur12, area12, marriage22, edu32, smoif22, drifre22, income42, gender2, work12,spotim2,year2)

dd2<- datadist(age53b)

options(datadist='dd2')

fit2 <- lrm(depress22~ rcs(ltpa2,3)+urb_rur12+area12 +marriage22+edu32+smoif22+drifre22+income42+gender2+work12+year2, data=age53b)

print(fit2)

dd2$limits$spotim2[2]<-0

fit2=update(fit2)

OR2<-Predict(fit2, ltpa2,fun=exp,ref.zero = TRUE)

p2<-ggplot(OR2)

anova(fit2)

rr<-ggplot()+geom_line(data=OR1, aes(ltpa2,yhat),linetype="solid",size=0.6,alpha = 0.3,colour="red")+

geom_ribbon(data=OR1, aes(ltpa2,ymin = lower, ymax = upper),alpha = 0.1,fill="red")+

geom_line(data=OR2, aes(ltpa2,yhat),linetype="solid",size=0.6,alpha = 0.7,colour="lightblue")+

geom_ribbon(data=OR2, aes(ltpa2,ymin = lower, ymax = upper),alpha = 0.5,fill="lightblue")+

geom_hline(yintercept=1, linetype=2,size=0.6)

rr1<-rr+ theme(axis.title.y=element_blank(),axis.text.y = element_blank())+

theme(panel.background = element_rect(fill = NA),

panel.grid.major.y = element_blank(),panel.grid.minor.y = element_blank(),

panel.grid.major.x = element_blank(),panel.grid.minor.x = element_blank(),

axis.line = element_line(size = 1, colour = "black"),

axis.text = element_text(size=6,colour = "black",family="A"),

axis.ticks = element_line(size = 1),

axis.ticks.length.y = unit(.15, "cm"),

axis.ticks.length.x = unit(.15, "cm"),

title=element_text(size=8, color="black"), legend.position = "none")+

scale_y_continuous(limits = c(0.35,1.0), expand = c(0.05,0),breaks = seq(0.4,1.0,0.2))+

scale_x_continuous(limits = c(0,2400), expand = c(0.05,0),breaks = seq(0,2400,600))+

labs( x="Total volume (MET-min/week)", y="OR (95%CI)")

rr2=rr1+guides(fill=F)

load("C:/Users/hustbim/Desktop/yangtiao/smoif21.RData")

depress22<-smoif21$depress2

ltpa2<-smoif21$ltpa1

urb_rur12<-smoif21$urb_rur

area12<-smoif21$area

marriage22<-smoif21$ marriage2

edu32<-smoif21$edu2

age2<-smoif21$age

drifre22<-smoif21$drifre2

work12<-smoif21$work1

income42<-smoif21$income2

gender2<-smoif21$gender

spotim2<-smoif21$spotim

year2<-smoif21$year

smoif21b<-data.frame(depress22,ltpa2, urb_rur12, area12, marriage22, edu32, age2, drifre22, income42, gender2,work12,spotim2,year2)

dd1<- datadist(smoif21b)

options(datadist='dd1')

fit1 <- lrm(depress22~ rcs(ltpa2,3)+urb_rur12+area12 +marriage22+edu32+age2+drifre22+income42+gender2+work12+year2, data=smoif21b)

print(fit1)

dd1$limits$spotim2[2]<-0

fit1=update(fit1)

OR1<-Predict(fit1, ltpa2,fun=exp,ref.zero = TRUE)

p1<-ggplot(OR1)

anova(fit1)

load("C:/Users/hustbim/Desktop/yangtiao/smoif22.RData")

depress22<-smoif22$depress2

ltpa2<-smoif22$ltpa1

urb_rur12<-smoif22$urb_rur

area12<-smoif22$area

marriage22<-smoif22$ marriage2

edu32<-smoif22$edu2

age2<-smoif22$age

drifre22<-smoif22$drifre2

work12<-smoif22$work1

income42<-smoif22$income2

gender2<-smoif22$gender

spotim2<-smoif22$spotim

year2<-smoif22$year

smoif22b<-data.frame(depress22,ltpa2, urb_rur12, area12, marriage22, edu32, age2, drifre22, income42, gender2, work12,spotim2,year2)

dd2<- datadist(smoif22b)

options(datadist='dd2')

fit2 <- lrm(depress22~ rcs(ltpa2,3)+urb_rur12+area12 +marriage22+edu32+age2+drifre22+income42+gender2+work12+year2, data=smoif22b)

print(fit2)

dd2$limits$spotim2[2]<-0

fit2=update(fit2)

OR2<-Predict(fit2, ltpa2,fun=exp,ref.zero = TRUE)

p2<-ggplot(OR2)

anova(fit2)

ss<-ggplot()+geom_line(data=OR1, aes(ltpa2,yhat),linetype="solid",size=0.6,alpha = 0.3,colour="red")+

geom_ribbon(data=OR1, aes(ltpa2,ymin = lower, ymax = upper),alpha = 0.1,fill="red")+

geom_line(data=OR2, aes(ltpa2,yhat),linetype="solid",size=0.6,alpha = 0.7,colour="lightblue")+

geom_ribbon(data=OR2, aes(ltpa2,ymin = lower, ymax = upper),alpha = 0.5,fill="lightblue")+

geom_hline(yintercept=1, linetype=2,size=0.6)

ss1<-ss+ theme(axis.title.y=element_blank(),axis.text.y = element_blank())+

theme(panel.background = element_rect(fill = NA),

panel.grid.major.y = element_blank(),panel.grid.minor.y = element_blank(),

panel.grid.major.x = element_blank(),panel.grid.minor.x = element_blank(),

axis.line = element_line(size = 1, colour = "black"),

axis.text = element_text(size=6,colour = "black",family="A"),

axis.ticks = element_line(size = 1),

axis.ticks.length.y = unit(.15, "cm"),

axis.ticks.length.x = unit(.15, "cm"),

title=element_text(size=8, color="black"), legend.position = "none")+

scale_y_continuous(limits = c(0.35,1.0), expand = c(0.05,0),breaks = seq(0.4,1.0,0.2))+

scale_x_continuous(limits = c(0,2400), expand = c(0.05,0),breaks = seq(0,2400,600))+

labs( x="Total volume (MET-min/week)", y="OR (95%CI)")

ss2=ss1+guides(fill=F)

load("C:/Users/hustbim/Desktop/yangtiao/drifre21.RData")

depress22<-drifre21$depress2

ltpa2<-drifre21$ltpa1

urb_rur12<-drifre21$urb_rur

area12<-drifre21$area

marriage22<-drifre21$ marriage2

edu32<-drifre21$edu2

smoif22<-drifre21$smoif2

age2<-drifre21$age

work12<-drifre21$work1

income42<-drifre21$income2

gender2<-drifre21$gender

spotim2<-drifre21$spotim

year2<-drifre21$year

drifre21b<-data.frame(depress22,ltpa2, urb_rur12, area12, marriage22, edu32, smoif22, age2, income42, gender2,work12,spotim2,year2)

dd1<- datadist(drifre21b)

options(datadist='dd1')

fit1 <- lrm(depress22~ rcs(ltpa2,3)+urb_rur12+area12 +marriage22+edu32+smoif22+age2+income42+gender2+work12+year2, data=drifre21b)

print(fit1)

dd1$limits$spotim2[2]<-0

fit1=update(fit1)

OR1<-Predict(fit1, ltpa2,fun=exp,ref.zero = TRUE)

p1<-ggplot(OR1)

anova(fit1)

load("C:/Users/hustbim/Desktop/yangtiao/drifre22.RData")

depress22<-drifre22$depress2

ltpa2<-drifre22$ltpa1

urb_rur12<-drifre22$urb_rur

area12<-drifre22$area

marriage22<-drifre22$ marriage2

edu32<-drifre22$edu2

smoif22<-drifre22$smoif2

age2<-drifre22$age

work12<-drifre22$work1

income42<-drifre22$income2

gender2<-drifre22$gender

spotim2<-drifre22$spotim

year2<-drifre22$year

drifre22b<-data.frame(depress22,ltpa2, urb_rur12, area12, marriage22, edu32, smoif22, age2, income42, gender2, work12,spotim2,year2)

dd2<- datadist(drifre22b)

options(datadist='dd2')

fit2 <- lrm(depress22~ rcs(ltpa2,3)+urb_rur12+area12 +marriage22+edu32+smoif22+age2+income42+gender2+work12+year2, data=drifre22b)

print(fit2)

dd2$limits$spotim2[2]<-0

fit2=update(fit2)

OR2<-Predict(fit2, ltpa2,fun=exp,ref.zero = TRUE)

p2<-ggplot(OR2)

anova(fit2)

tt<-ggplot()+geom_line(data=OR1, aes(ltpa2,yhat),linetype="solid",size=0.6,alpha = 0.3,colour="red")+

geom_ribbon(data=OR1, aes(ltpa2,ymin = lower, ymax = upper),alpha = 0.1,fill="red")+

geom_line(data=OR2, aes(ltpa2,yhat),linetype="solid",size=0.6,alpha = 0.7,colour="lightblue")+

geom_ribbon(data=OR2, aes(ltpa2,ymin = lower, ymax = upper),alpha = 0.5,fill="lightblue")+

geom_hline(yintercept=1, linetype=2,size=0.6)

tt1<-tt+ theme(axis.title.y=element_blank(),axis.text.y = element_blank())+

theme(panel.background = element_rect(fill = NA),

panel.grid.major.y = element_blank(),panel.grid.minor.y = element_blank(),

panel.grid.major.x = element_blank(),panel.grid.minor.x = element_blank(),

axis.line = element_line(size = 1, colour = "black"),

axis.text = element_text(size=6,colour = "black",family="A"),

axis.ticks = element_line(size = 1),

axis.ticks.length.y = unit(.15, "cm"),

axis.ticks.length.x = unit(.15, "cm"),

title=element_text(size=8, color="black"), legend.position = "none")+

scale_y_continuous(limits = c(0.35,1.0), expand = c(0.05,0),breaks = seq(0.4,1.0,0.2))+

scale_x_continuous(limits = c(0,2400), expand = c(0.05,0),breaks = seq(0,2400,600))+

labs( x="Total volume (MET-min/week)", y="OR (95%CI)")

tt2=tt1+guides(fill=F)

load("C:/Users/hustbim/Desktop/yangtiao/manbing10.RData")

depress22<-manbing10$depress2

ltpa2<-manbing10$ltpa1

urb_rur12<-manbing10$urb_rur

area12<-manbing10$area

marriage22<-manbing10$ marriage2

edu32<-manbing10$edu2

smoif22<-manbing10$smoif2

drifre22<-manbing10$drifre2

work12<-manbing10$work1

income42<-manbing10$income2

gender2<-manbing10$gender

spotim2<-manbing10$spotim

age2<-manbing10$age

year2<-manbing10$year2

manbing10b<-data.frame(depress22,ltpa2, urb_rur12, area12, marriage22, edu32, smoif22, drifre22, income42, gender2,work12,spotim2,age2,year2)

dd1<- datadist(manbing10b)

options(datadist='dd1')

fit1 <- lrm(depress22~ rcs(ltpa2,3)+urb_rur12+area12 +marriage22+edu32+smoif22+drifre22+income42+gender2+work12+age2+year2, data=manbing10b)

print(fit1)

dd1$limits$spotim2[2]<-0

fit1=update(fit1)

OR1<-Predict(fit1, ltpa2,fun=exp,ref.zero = TRUE)

p1<-ggplot(OR1)

anova(fit1)

load("C:/Users/hustbim/Desktop/yangtiao/manbing11.RData")

depress22<-manbing11$depress2

ltpa2<-manbing11$ltpa1

urb_rur12<-manbing11$urb_rur

area12<-manbing11$area

marriage22<-manbing11$ marriage2

edu32<-manbing11$edu2

smoif22<-manbing11$smoif2

drifre22<-manbing11$drifre2

work12<-manbing11$work1

income42<-manbing11$income2

gender2<-manbing11$gender

spotim2<-manbing11$spotim

age2<-manbing11$age

year2<-manbing11$year

manbing11b<-data.frame(depress22,ltpa2, urb_rur12, area12, marriage22, edu32, smoif22, drifre22, income42, gender2, work12,spotim2,age2,year2)

dd2<- datadist(manbing11b)

options(datadist='dd2')

fit2 <- lrm(depress22~ rcs(ltpa2,3)+urb_rur12+area12 +marriage22+edu32+smoif22+drifre22+income42+gender2+work12+age2+year2, data=manbing11b)

print(fit2)

dd2$limits$spotim2[2]<-0

fit2=update(fit2)

OR2<-Predict(fit2, ltpa2,fun=exp,ref.zero = TRUE)

p2<-ggplot(OR2)

anova(fit2)

uu<-ggplot()+geom_line(data=OR1, aes(ltpa2,yhat),linetype="solid",size=0.6,alpha = 0.3,colour="red")+

geom_ribbon(data=OR1, aes(ltpa2,ymin = lower, ymax = upper),alpha = 0.1,fill="red")+

geom_line(data=OR2, aes(ltpa2,yhat),linetype="solid",size=0.6,alpha = 0.7,colour="lightblue")+

geom_ribbon(data=OR2, aes(ltpa2,ymin = lower, ymax = upper),alpha = 0.5,fill="lightblue")+

geom_hline(yintercept=1, linetype=2,size=0.6)

uu1<-uu+ theme(axis.title.y=element_blank(),axis.text.y = element_blank())+

theme(panel.background = element_rect(fill = NA),

panel.grid.major.y = element_blank(),panel.grid.minor.y = element_blank(),

panel.grid.major.x = element_blank(),panel.grid.minor.x = element_blank(),

axis.line = element_line(size = 1, colour = "black"),

axis.text = element_text(size=6,colour = "black",family="A"),

axis.ticks = element_line(size = 1),

axis.ticks.length.y = unit(.15, "cm"),

axis.ticks.length.x = unit(.15, "cm"),

title=element_text(size=8, color="black"), legend.position = "none")+

scale_y_continuous(limits = c(0.35,1.0), expand = c(0.05,0),breaks = seq(0.4,1.0,0.2))+

scale_x_continuous(limits = c(0,2400), expand = c(0.05,0),breaks = seq(0,2400,600))+

labs( x="Total volume (MET-min/week)", y="OR (95%CI)")

uu2=uu1+guides(fill=F)

grid.arrange(mm2,rr2,ss2,tt2,uu2, ncol=5, nrow =1)
