# Supplementary material for: Large-scale data reveal disparate associations between leisure time physical activity patterns and mental health
Source: Commun Med (Lond). 2023 Dec 21;3:175. doi: 10.1038/s43856-023-00399-2 (PMC10739930; doi:10.1038/s43856-023-00399-2)
Supplement: Supplementary file 9 — Reporting Summary [file 43856_2023_399_MOESM9_ESM.pdf]

nature portfolio

Corresponding author(s): Lieyun Ding
Last updated by author(s): Jul 24, 2023

Reporting Summary

Nature Portfolio wishes to improve the reproducibility of the work that we publish. This form provides structure for consistency and transparency in reporting. For further information on Nature Portfolio policies, see our Editorial Policies and the Editorial Policy Checklist.

Statistics

For all statistical analyses, confirm that the following items are present in the figure legend, table legend, main text, or Methods section.

n/a	Confirmed	
☐	☒	The exact sample size (*n*) for each experimental group/condition, given as a discrete number and unit of measurement
☐	☒	A statement on whether measurements were taken from distinct samples or whether the same sample was measured repeatedly
☒	☐	The statistical test(s) used AND whether they are one- or two-sided<br>*Only common tests should be described solely by name; describe more complex techniques in the Methods section.*
☐	☒	A description of all covariates tested
☐	☒	A description of any assumptions or corrections, such as tests of normality and adjustment for multiple comparisons
☐	☒	A full description of the statistical parameters including central tendency (e.g. means) or other basic estimates (e.g. regression coefficient) AND variation (e.g. standard deviation) or associated estimates of uncertainty (e.g. confidence intervals)
☐	☒	For null hypothesis testing, the test statistic (e.g. *F*, *t*, *r*) with confidence intervals, effect sizes, degrees of freedom and *P* value noted<br>*Give P values as exact values whenever suitable.*
☒	☐	For Bayesian analysis, information on the choice of priors and Markov chain Monte Carlo settings
☒	☐	For hierarchical and complex designs, identification of the appropriate level for tests and full reporting of outcomes
☒	☐	Estimates of effect sizes (e.g. Cohen's *d*, Pearson's *r*), indicating how they were calculated

Our web collection on statistics for biologists contains articles on many of the points above.

Software and code

Policy information about availability of computer code

Data collection	Data tenure was controlled by Centre for Health Statistics Information, National Health Commission of the People's Republic of China. Raw data are not publicly available due to privacy considerations. Data access can be requested by email to the corresponding author.
Data analysis	Stata and R codes for the statistical models in the main-text are documented as the Supplementary Software. All codes are available from the corresponding author upon request. All analyses used Stata 16.0 or R 3.5.1.

For manuscripts utilizing custom algorithms or software that are central to the research but not yet described in published literature, software must be made available to editors and reviewers. We strongly encourage code deposition in a community repository (e.g. GitHub). See the Nature Portfolio guidelines for submitting code & software for further information.

Data

Policy information about availability of data

All manuscripts must include a data availability statement. This statement should provide the following information, where applicable:

- Accession codes, unique identifiers, or web links for publicly available datasets
- A description of any restrictions on data availability
- For clinical datasets or third party data, please ensure that the statement adheres to our policy

Data tenure was controlled by Centre for Health Statistics Information, National Health Commission of the People's Republic of China. Raw data are not publicly

available due to privacy considerations. Data access can be requested by email to the corresponding author. The source data for Figures 1, 2, and Supplementary Figure 3 can be found in Supplementary Data 2, 3, and 4 respectively.

Research involving human participants, their data, or biological material

Policy information about studies with human participants or human data. See also policy information about sex, gender (identity/presentation), and sexual orientation and race, ethnicity and racism.

Reporting on sex and gender	A total 711,759 individuals were included in this study, among whom 351,853 (49.43%) were male and 359,906 (50.57%) were female.
Reporting on race, ethnicity, or other socially relevant groupings	Age was calculated according to birth date and classified into three groups (15-29 years, 30-59 years, and 60 years or above) Gender was expressed as a binary variable (female or male). Smoking was defined as one had smoked a total of at least 100 cigarettes and had not quit smoking and smoking status was dichotomized as "smoker" or "non-smoker". Alcohol consumption was defined as having had an alcoholic drink in the 12 months before the survey and was dichotomized as "drinker" or "non- drinker". Income level was classified into three groups (low, middle, and high) based on annual per capital income in the sampled county at the survey year. Education level was categorized into four groups (college or above, high school, middle school, and primary school or below). Occupation status was classified into four groups (employed, retired, student, and unemployed). Marital status was categorized into four groups (married, unmarried, divorced, and widowed). Urbanization (urban or rural) and geographical regions (east, central, or west) and were considered on the basis of residential address. Year (2003, 2008, 2013, and 2018) was also adjusted in all models.
Population characteristics	Individuals aged 15-29 years were 19.36%, aged 30-59 years were 56.47%, and aged 60 years or above were 24.17%. Of the 711759 individuals, a greater proportion were with a primary school or lower level of education (39.43%), middle level of income (50.01%), and employed (65.78%). The majority of respondents did not consume alcohol (80.63%) or smoke (71.32%) and with no chronic disease (74.78%).
Recruitment	Our study based on the latest four rounds (2003, 2008, 2013 and 2018) of the National Health Services Survey (NHSS) covering all 31 provinces, autonomous regions, and municipalities in the mainland of China. The NHSS has been organized by the National Health Commission (NHC) of the People's Republic of China every fifth year since 1993. It uses multistage stratified cluster sampling. Specifically, the mainland of China was divided into east, central, and west regions and then sampled counties from each region stratified by urban and rural areas. To cover 0.02% of the population of the whole country and take into account a 2% non-respondent rate, at least 90 counties and 600 households for each county were needed to be sampled. In stage one, 95 of a possible 2861 counties were randomly selected in 2003, and 94 of which were chosen again in the 2008 survey (one county was excluded because its administrative division had changed). To better represent the increased urbanisation of China, another 52 counties from urban areas and 10 counties from rural areas were randomly selected in addition to those counties involved in the 2008 survey (a total of 156 counties sampled) in 2013 and 2018. In stage two, five streets in urban areas or townships in rural areas from each county were selected at random. In stage three, two urban communities from each urban street or rural villages from each rural township were sampled respectively. In stage four, 60 households were randomly sampled in each administrative community or village (each of which would typically contain 500–3000 households). All respondents aged 15 years or older in the selected household were eligible in this investigation, and questions about children younger than 15 years were answered by adult family members.
Ethics oversight	The institutional review board of the Chinese National Bureau of Statistics provided approval of the survey.

Note that full information on the approval of the study protocol must also be provided in the manuscript.

Field-specific reporting

Please select the one below that is the best fit for your research. If you are not sure, read the appropriate sections before making your selection.

☐ Life sciences ☒ Behavioural & social sciences ☐ Ecological, evolutionary & environmental sciences

For a reference copy of the document with all sections, see nature.com/documents/nr-reporting-summary-flat.pdf

Behavioural & social sciences study design

All studies must disclose on these points even when the disclosure is negative.

Study description	This study is a quantitative cross-sectional study.
Research sample	Our study based on the National Health Services Survey (NHSS), which is one of the largest nationally representative cross-sectional household survey of Chinese residents' demographic and socioeconomic status, health insurance enrolment, health needs, health service access and utilisation, medical expenditures and health. The NHSS covers all 31 provinces, autonomous regions, and municipalities in the mainland of China.
Sampling strategy	The NHSS uses multistage stratified cluster sampling. Specifically, the mainland of China was divided into east, central, and west regions and then sampled counties from each region stratified by urban and rural areas. To cover 0.02% of the population of the whole country and take into account a 2% non-respondent rate, at least 90 counties and 600 households for each county were needed to be sampled. In stage one, 95 of a possible 2861 counties were randomly selected in 2003, and 94 of which were chosen again in the 2008 survey (one county was excluded because its administrative division had changed). To better represent the

	increased urbanisation of China, another 52 counties from urban areas and 10 counties from rural areas were randomly selected in addition to those counties involved in the 2008 survey (a total of 156 counties sampled) in 2013 and 2018. In stage two, five streets in urban areas or townships in rural areas from each county were selected at random. In stage three, two urban communities from each urban street or rural villages from each rural township were sampled respectively. In stage four, 60 households were randomly sampled in each administrative community or village (each of which would typically contain 500–3000 households).
Data collection	Based on a structured questionnaire, face-to-face interviews were conducted by local trained health-care workers including information about participants' demographic, socioeconomic, lifestyle behaviors (e.g., alcohol consumption, smoking, and LTPA), and health conditions.
Timing	Our study based on the latest four rounds (2003, 2008, 2013 and 2018) of the NHSS, which has been organized by the National Health Commission (NHC) of the People's Republic of China every fifth year since 1993.
Data exclusions	Across these four years, data relating to leisure time physical activity were available for 98% of individuals aged 15 years or older. We then excluded individuals who were missing response data on self-reported mental health burden over the last month (0.19%), gender (0.01%), smoking (1.45%), alcohol consumption (0.46%), self-reported chronic diseases (0.08%), education level (0.04%), occupation (0.06%), income level (1.46%).
Non-participation	43121 individuals were excluded in 2003, 37514 individuals were excluded in 2008, 57683 individuals were excluded in 2013, 51105 individuals were excluded in 2018.
Randomization	Participants were not allocated into experimental groups.

Reporting for specific materials, systems and methods

We require information from authors about some types of materials, experimental systems and methods used in many studies. Here, indicate whether each material, system or method listed is relevant to your study. If you are not sure if a list item applies to your research, read the appropriate section before selecting a response.

Materials & experimental systems

n/a	Involved in the study
☒	☐ Antibodies
☒	☐ Eukaryotic cell lines
☒	☐ Palaeontology and archaeology
☒	☐ Animals and other organisms
☒	☐ Clinical data
☒	☐ Dual use research of concern
☒	☐ Plants

Methods

n/a	Involved in the study
☒	☐ ChIP-seq
☒	☐ Flow cytometry
☒	☐ MRI-based neuroimaging
